# Supplementary figures and images for: Outer membrane protein N expressed in Gram-negative bacterial strain of Escherichia coli BL21 (DE3) Omp8 Rosetta strains under osmoregulation by salts, sugars, and pHs
Source: PLoS One. 2023 Aug 3;18(8):e0288096. doi: 10.1371/journal.pone.0288096 (PMC10399875; doi:10.1371/journal.pone.0288096)

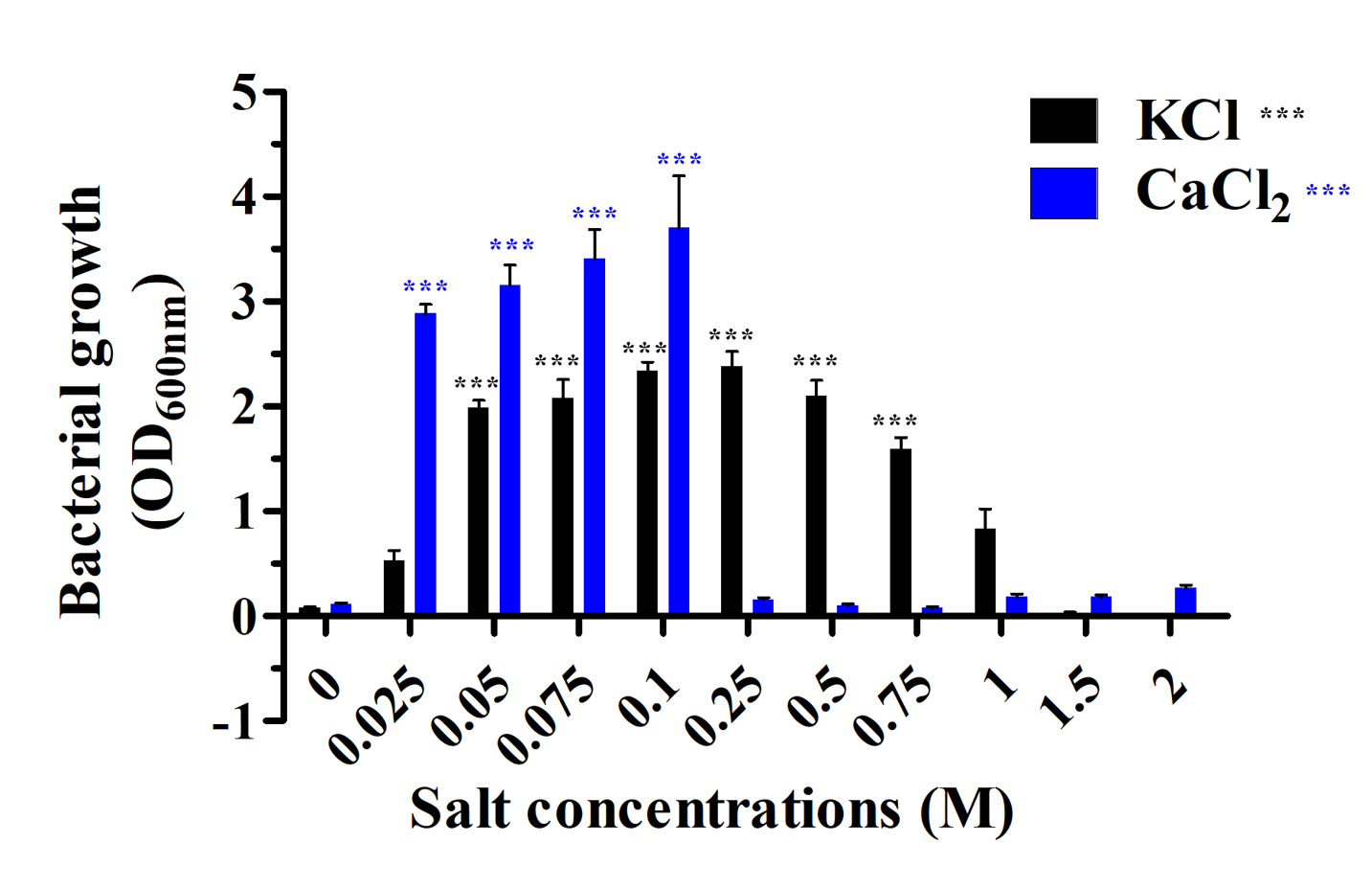

Supplement: S1 Fig — It is important to note that all of the data came from three different experiments. (TIF) [file pone.0288096.s001.tif]
