# Supplementary material for: Outer membrane protein N expressed in Gram-negative bacterial strain of Escherichia coli BL21 (DE3) Omp8 Rosetta strains under osmoregulation by salts, sugars, and pHs
Source: PLoS One. 2023 Aug 3;18(8):e0288096. doi: 10.1371/journal.pone.0288096 (PMC10399875; doi:10.1371/journal.pone.0288096)
Supplement: S1 File — (PPTX) [file pone.0288096.s002.pptx]

## Slide 1
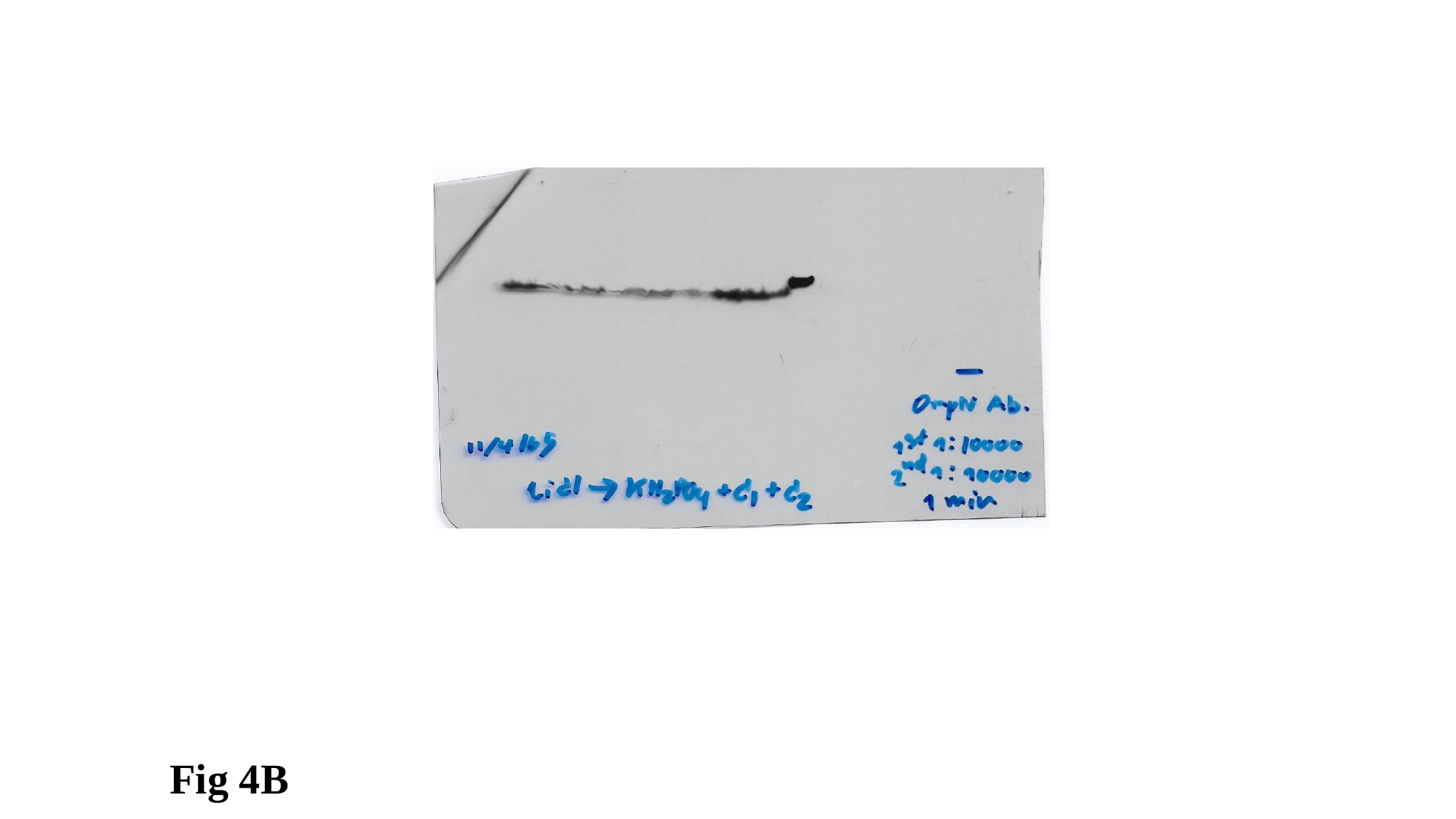

Fig 4B

## Slide 2
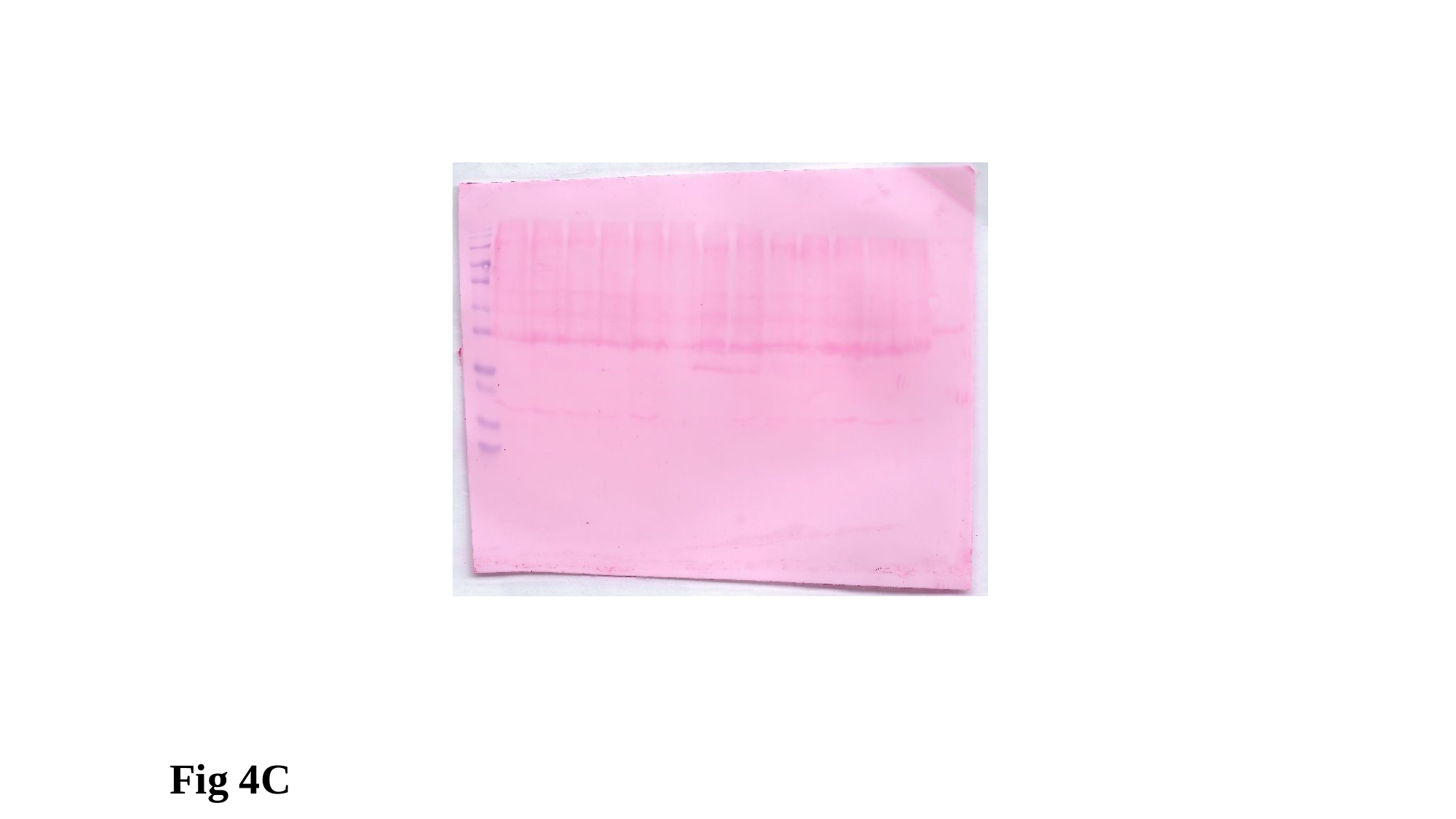

Fig 4C

## Slide 3
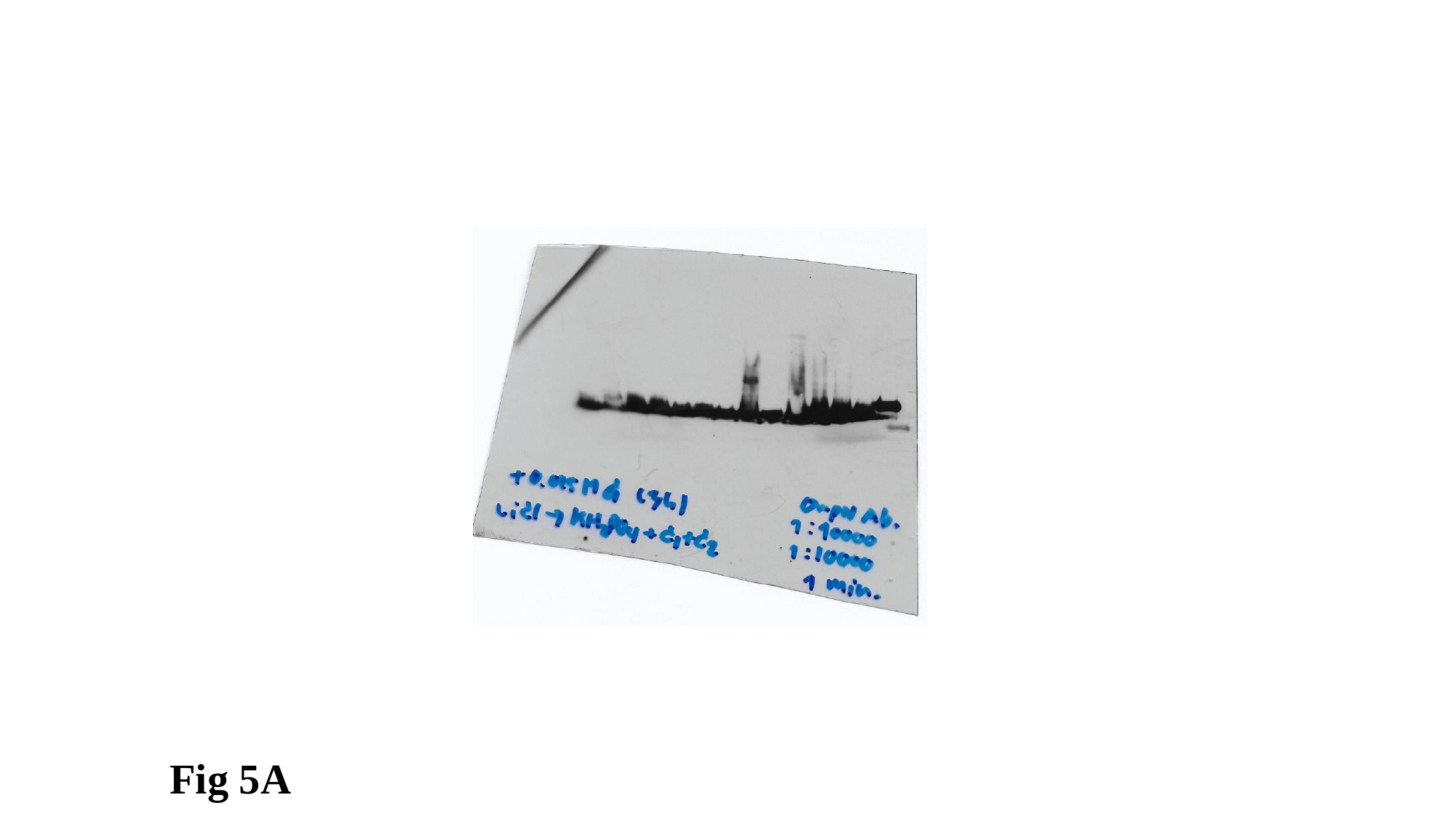

Fig 5A

## Slide 4
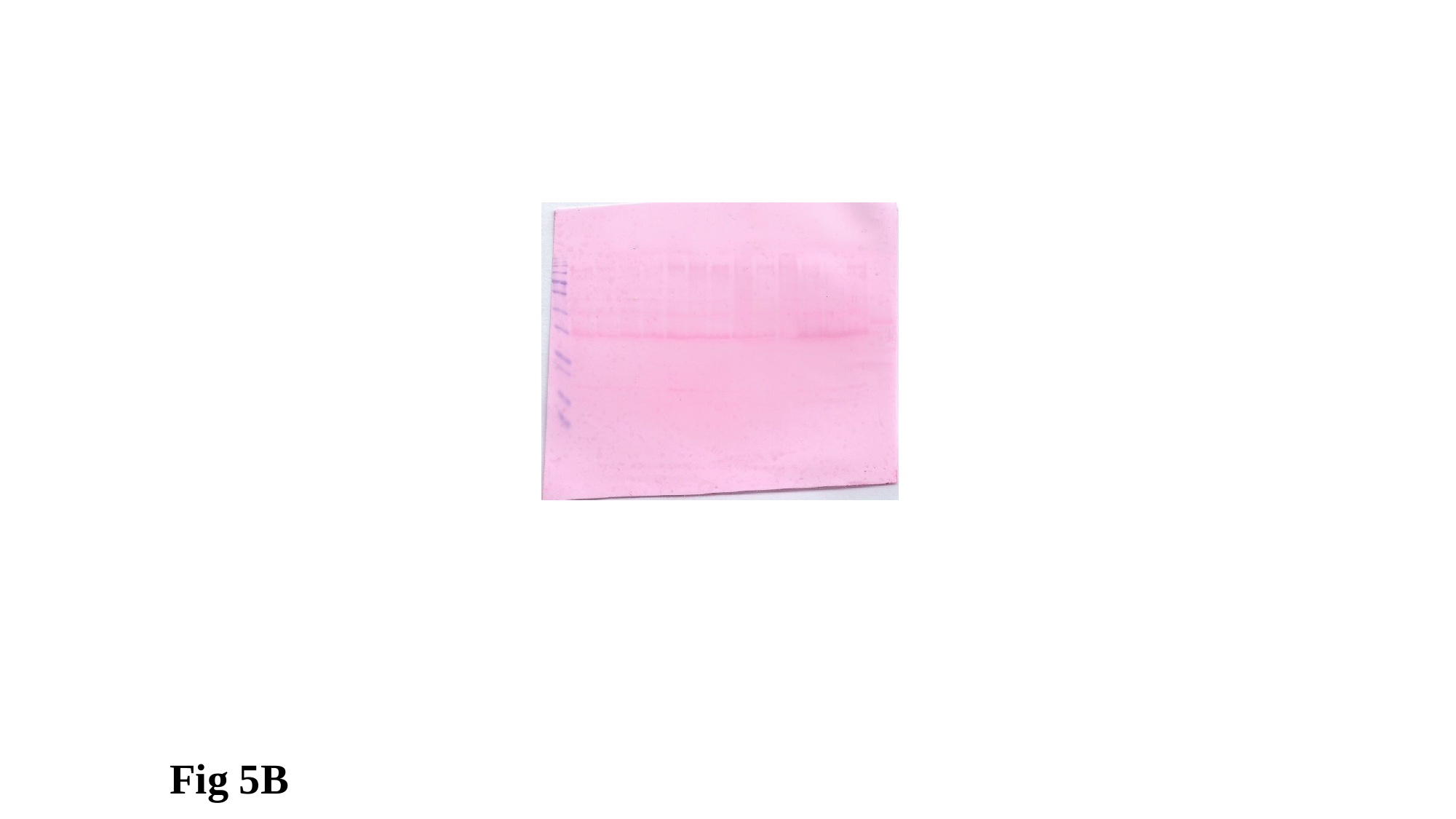

Fig 5B

## Slide 5
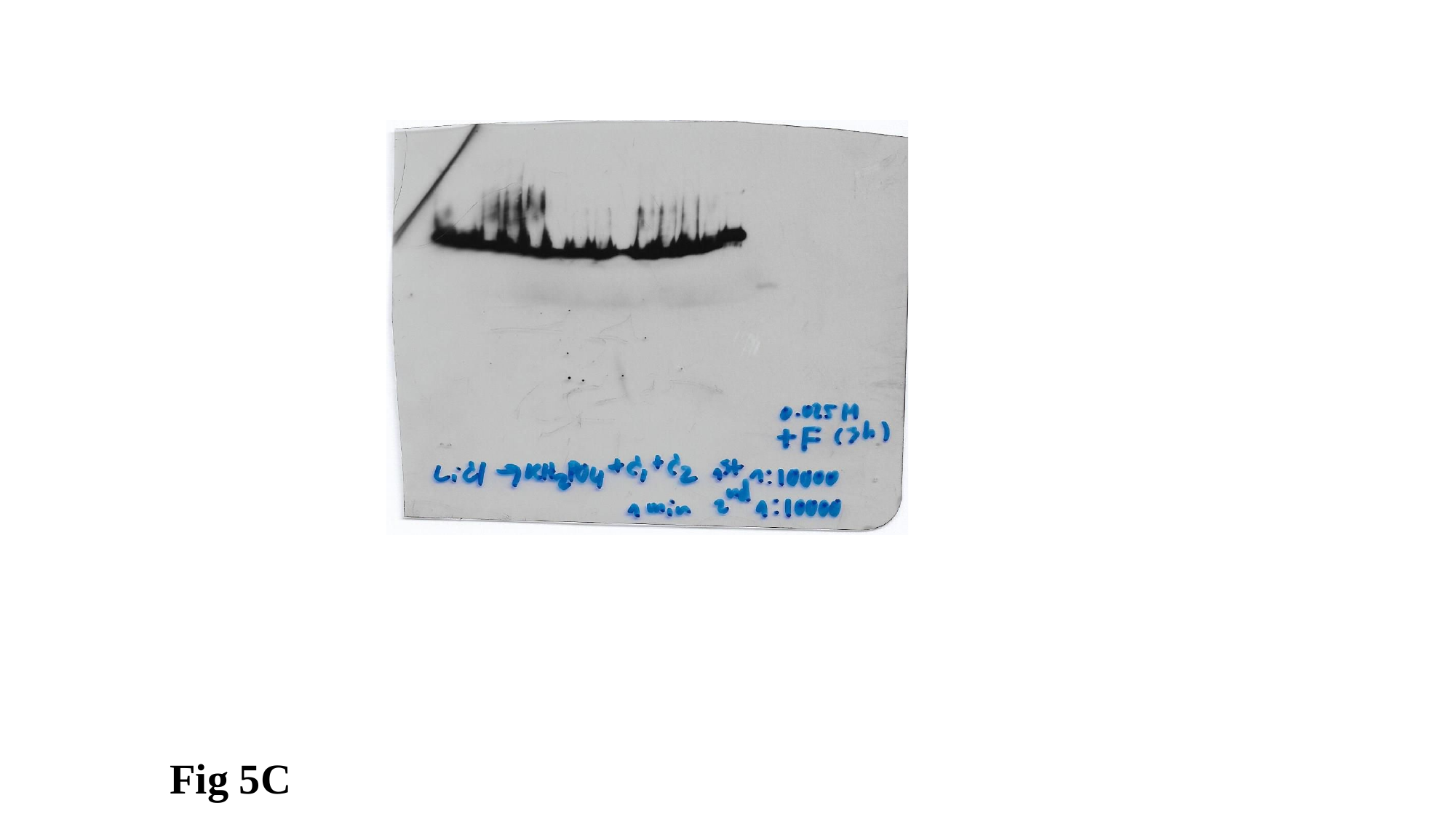

Fig 5C

## Slide 6
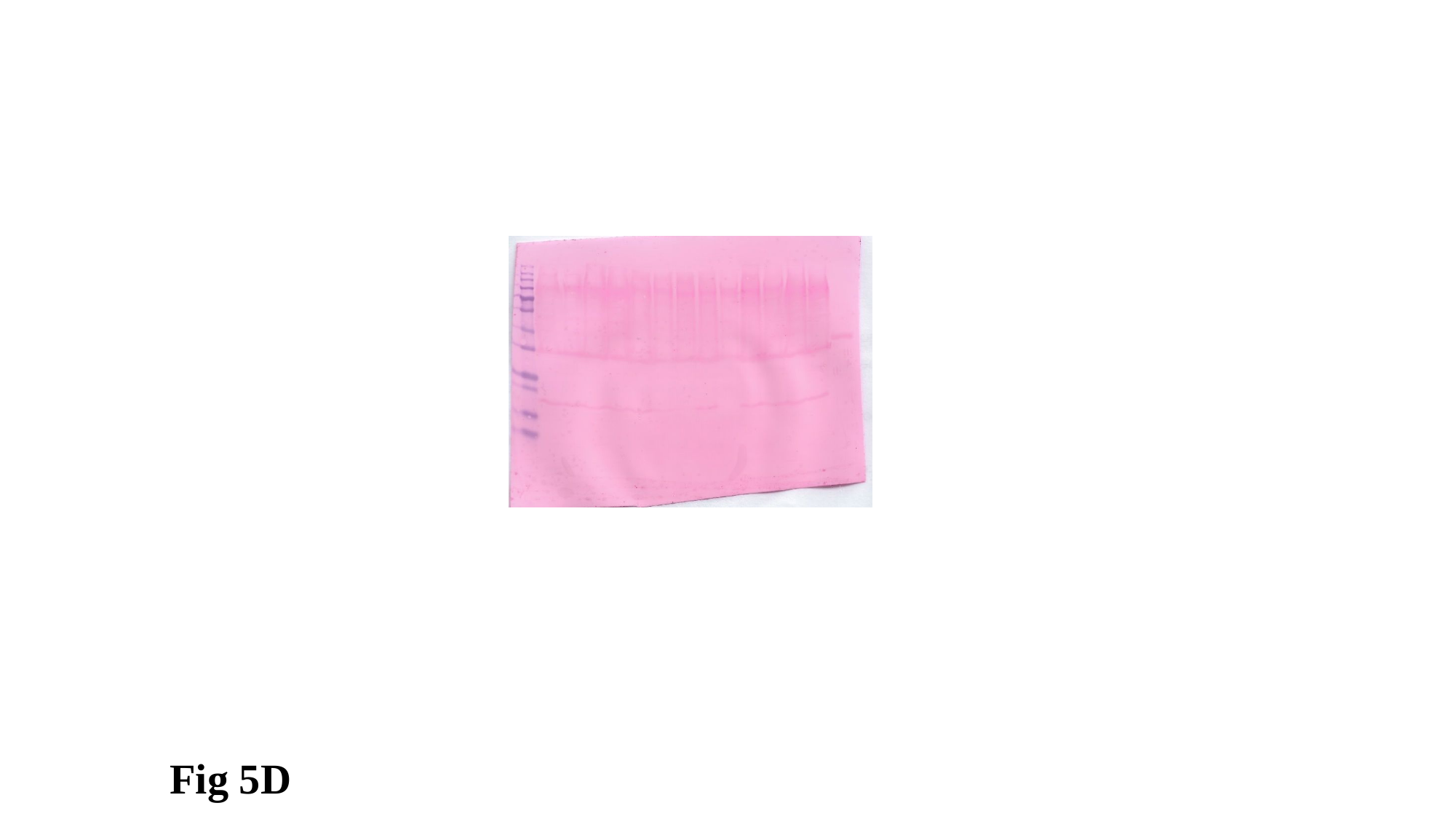

Fig 5D

## Slide 7
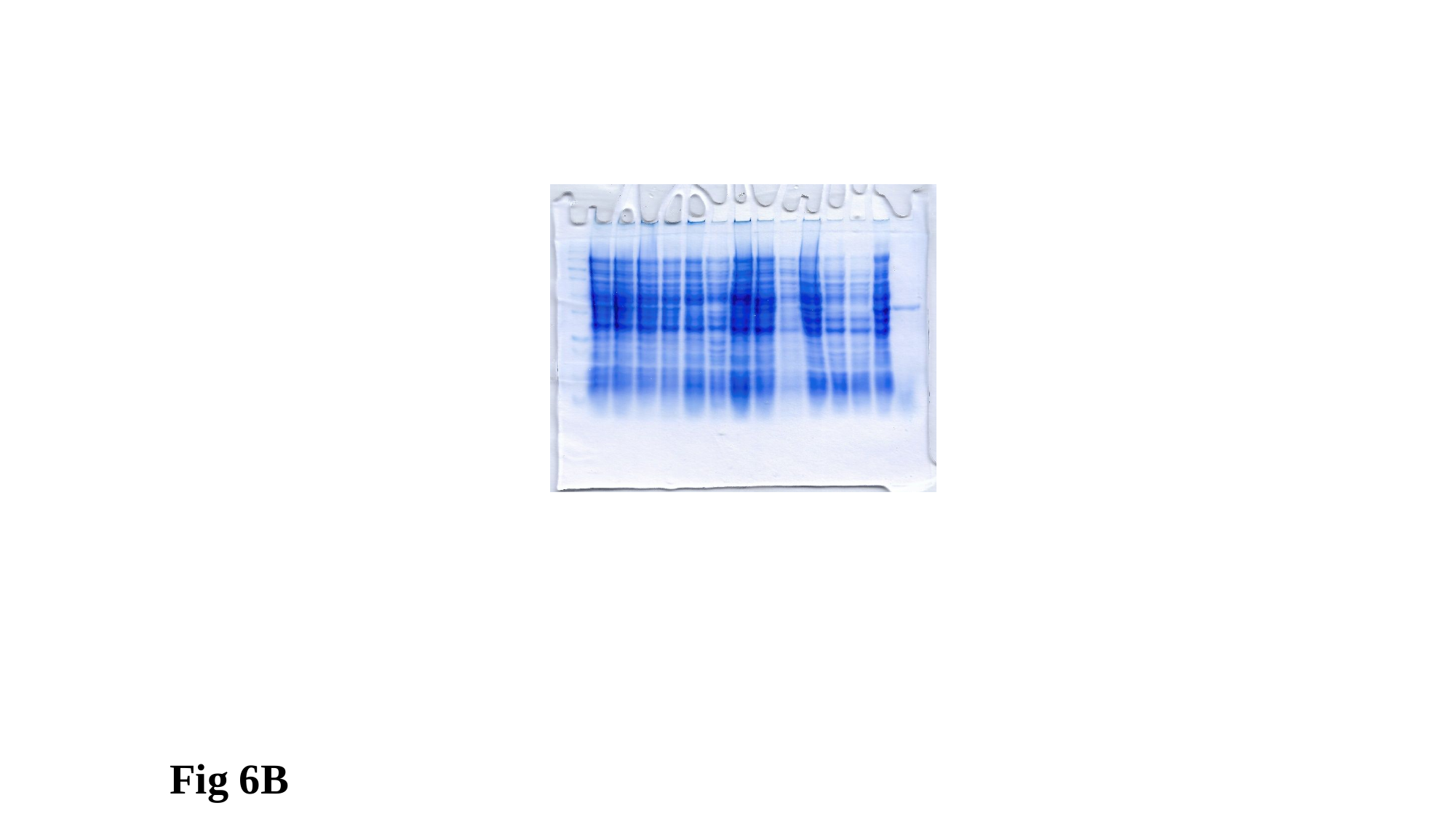

Fig 6B

## Slide 8
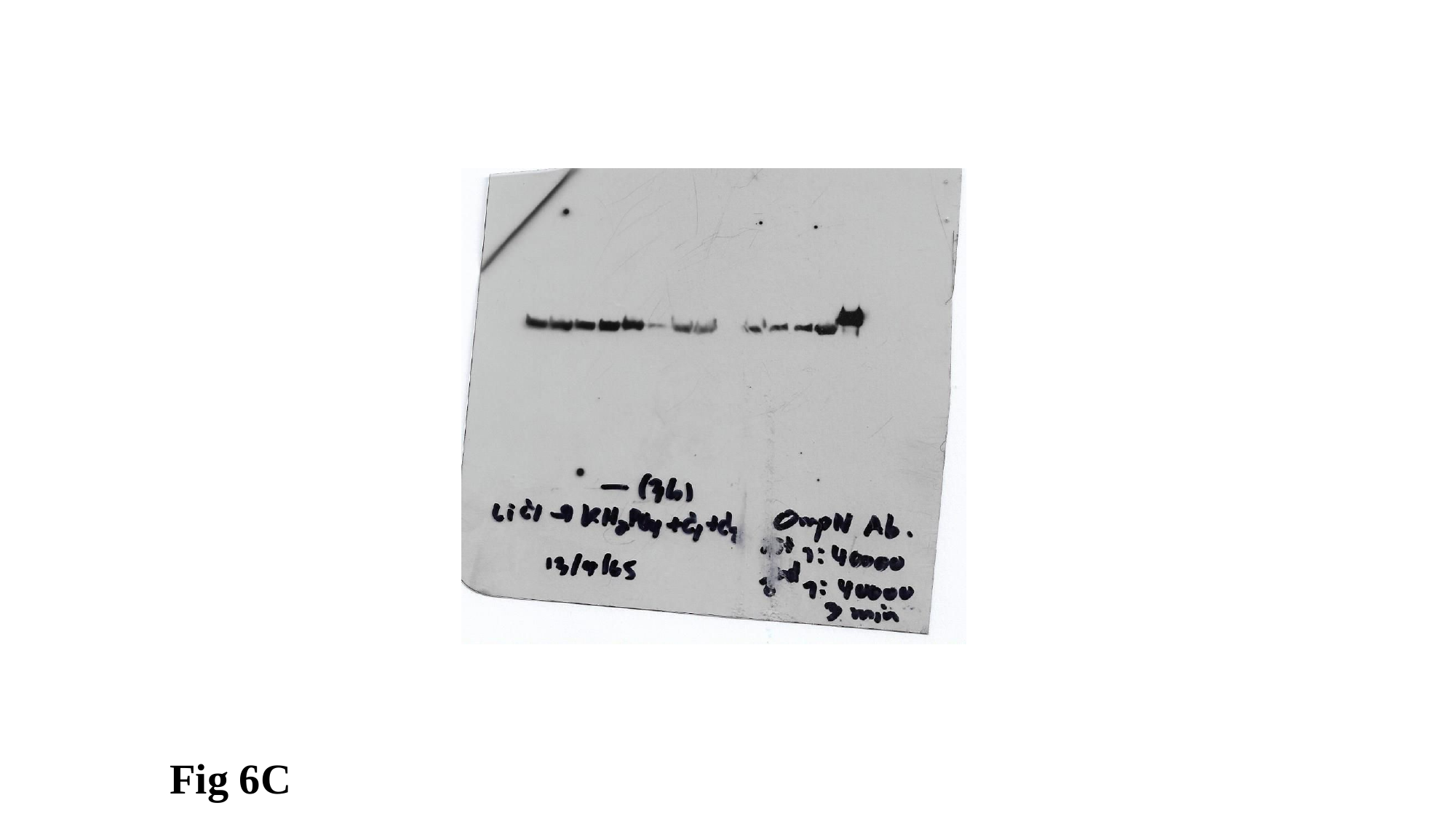

Fig 6C

## Slide 9
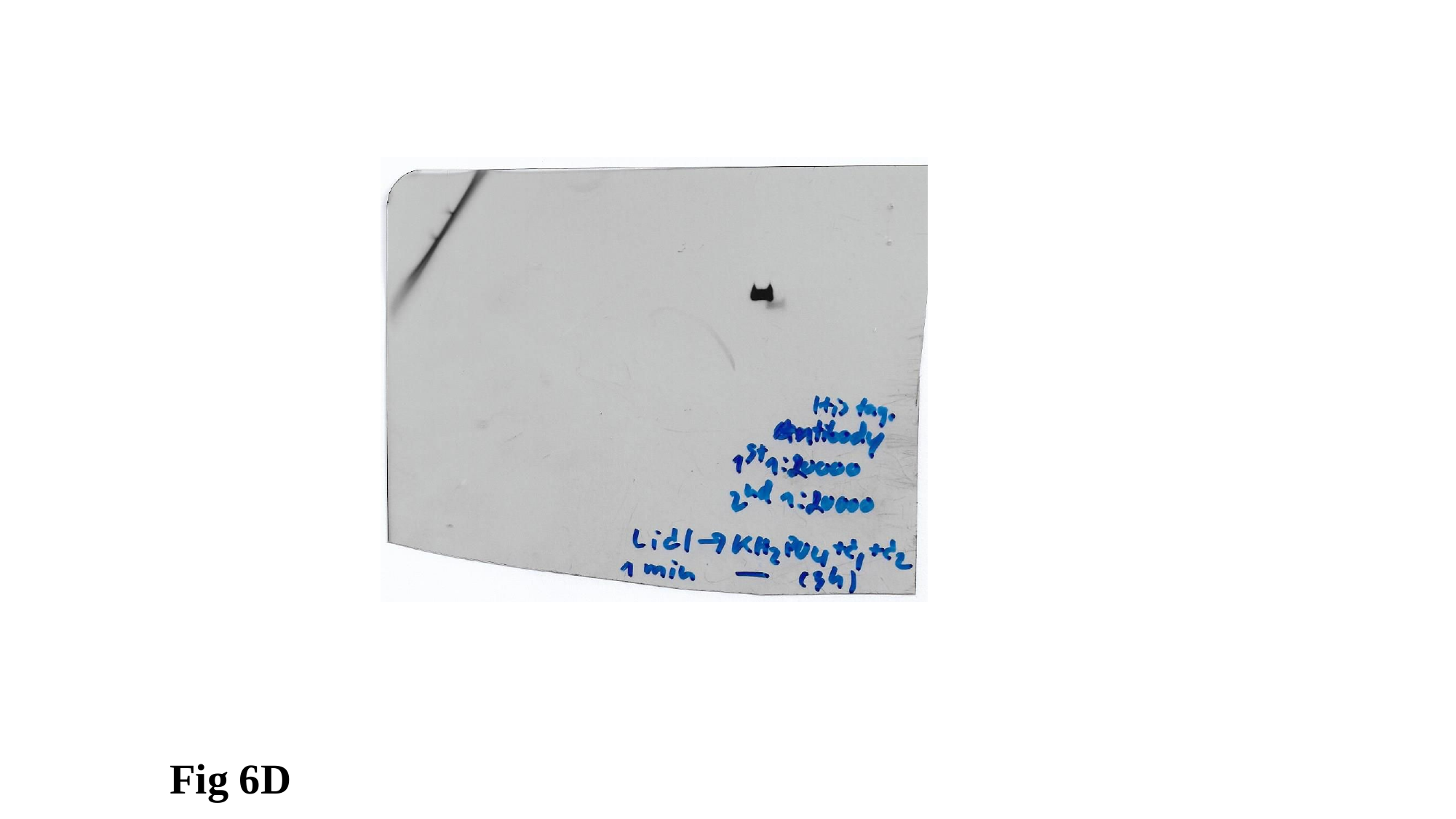

Fig 6D

## Slide 10
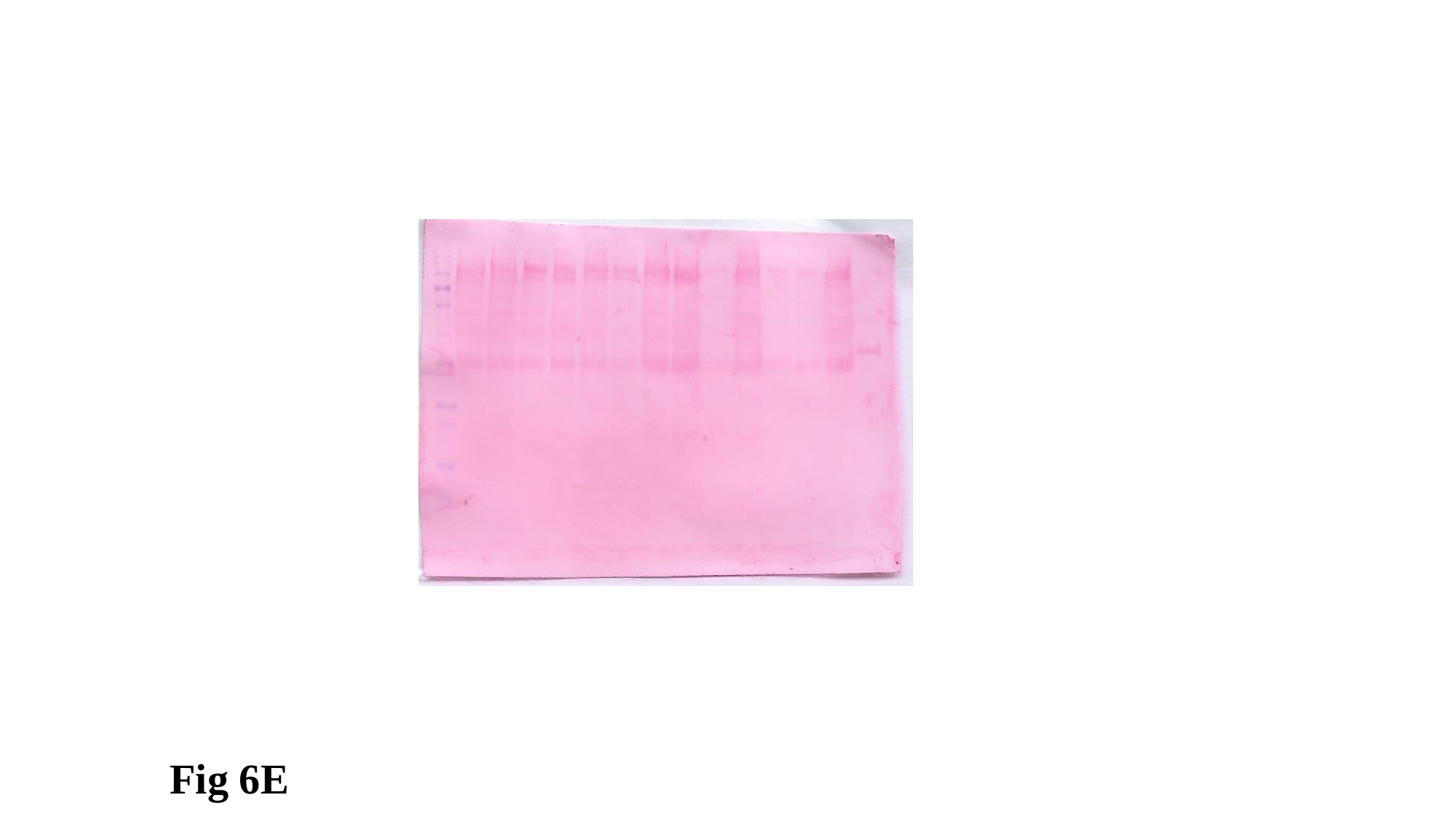

Fig 6E

## Slide 11
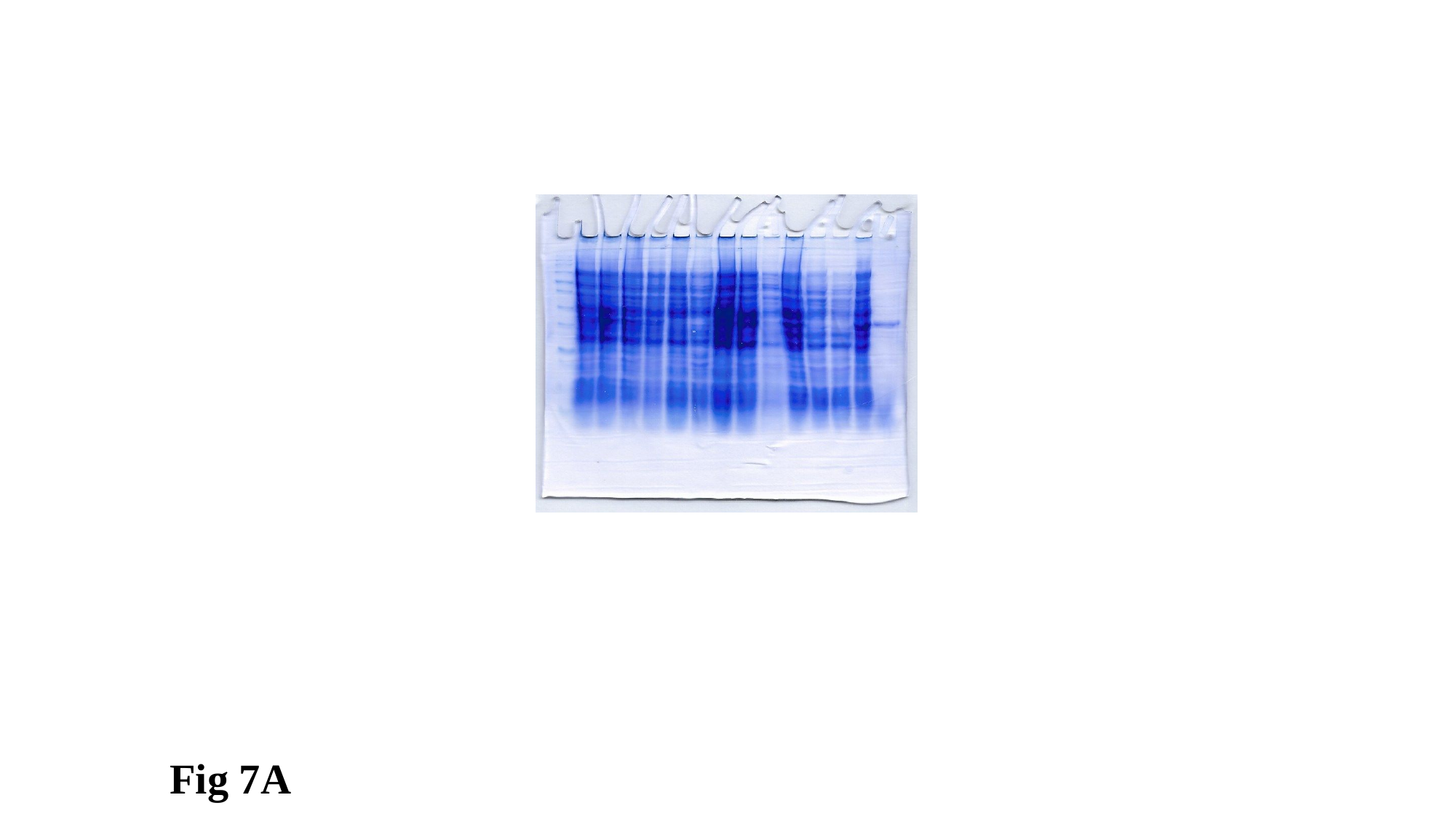

Fig 7A

## Slide 12
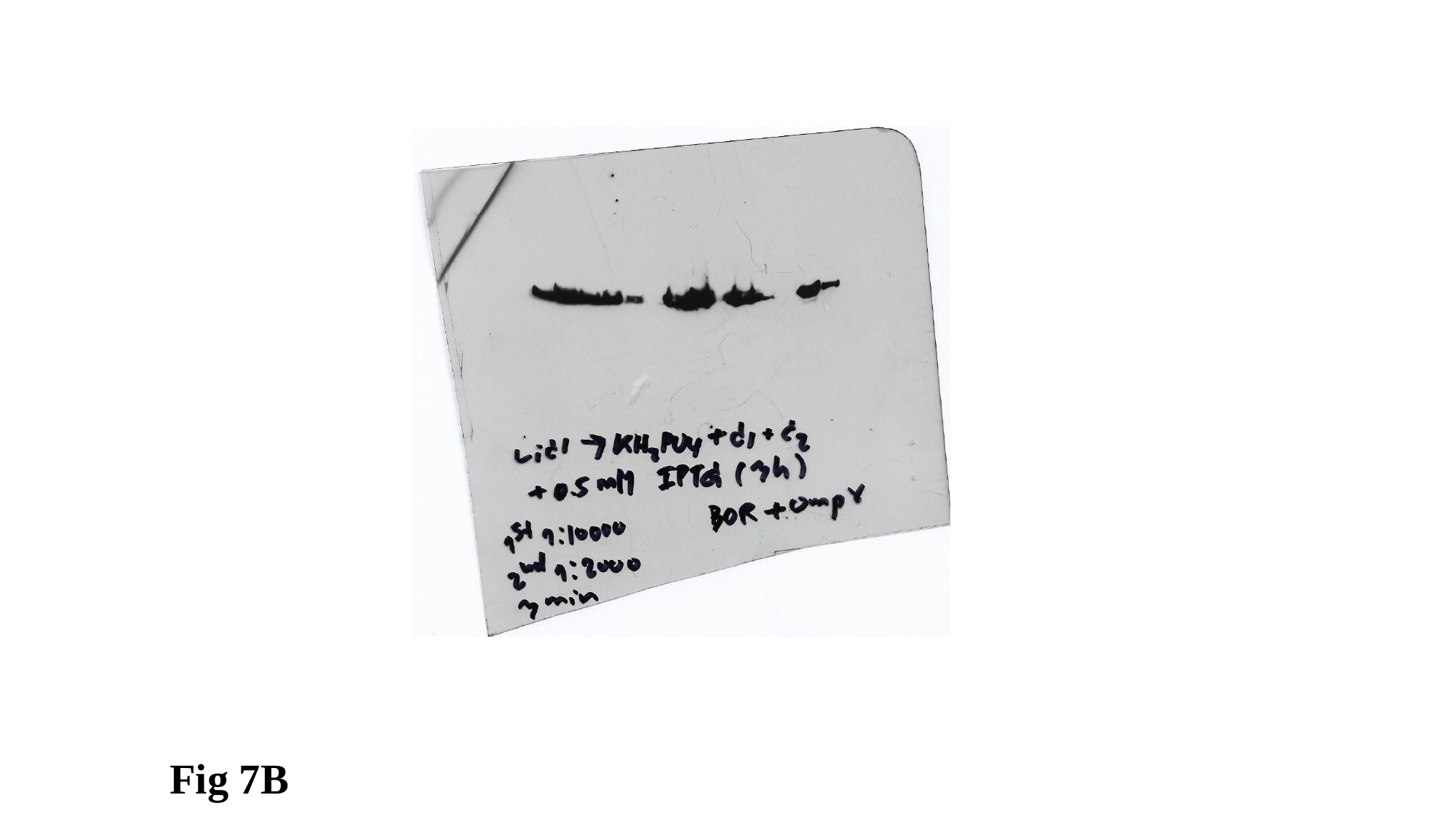

Fig 7B

## Slide 13
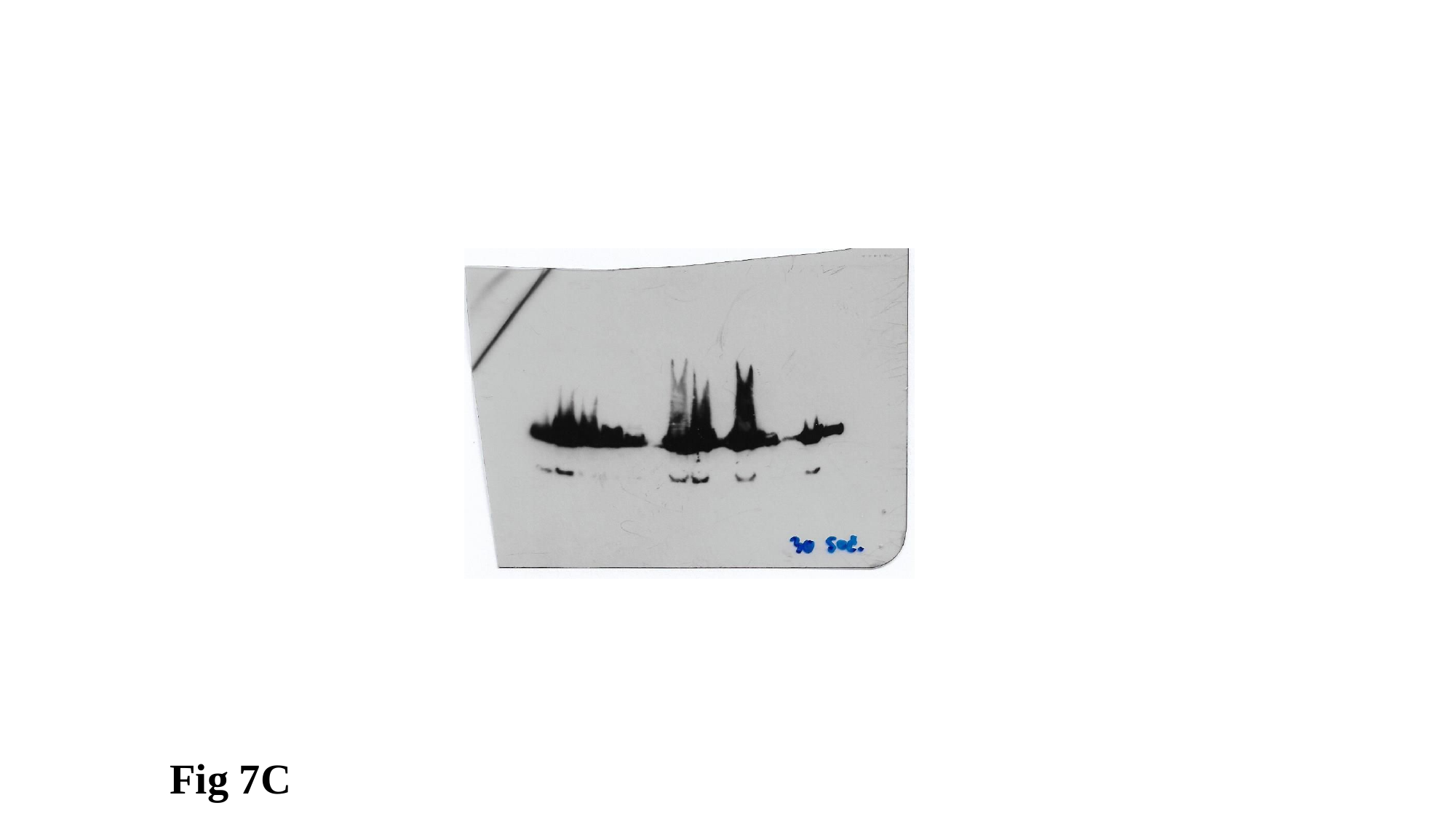

Fig 7C

## Slide 14
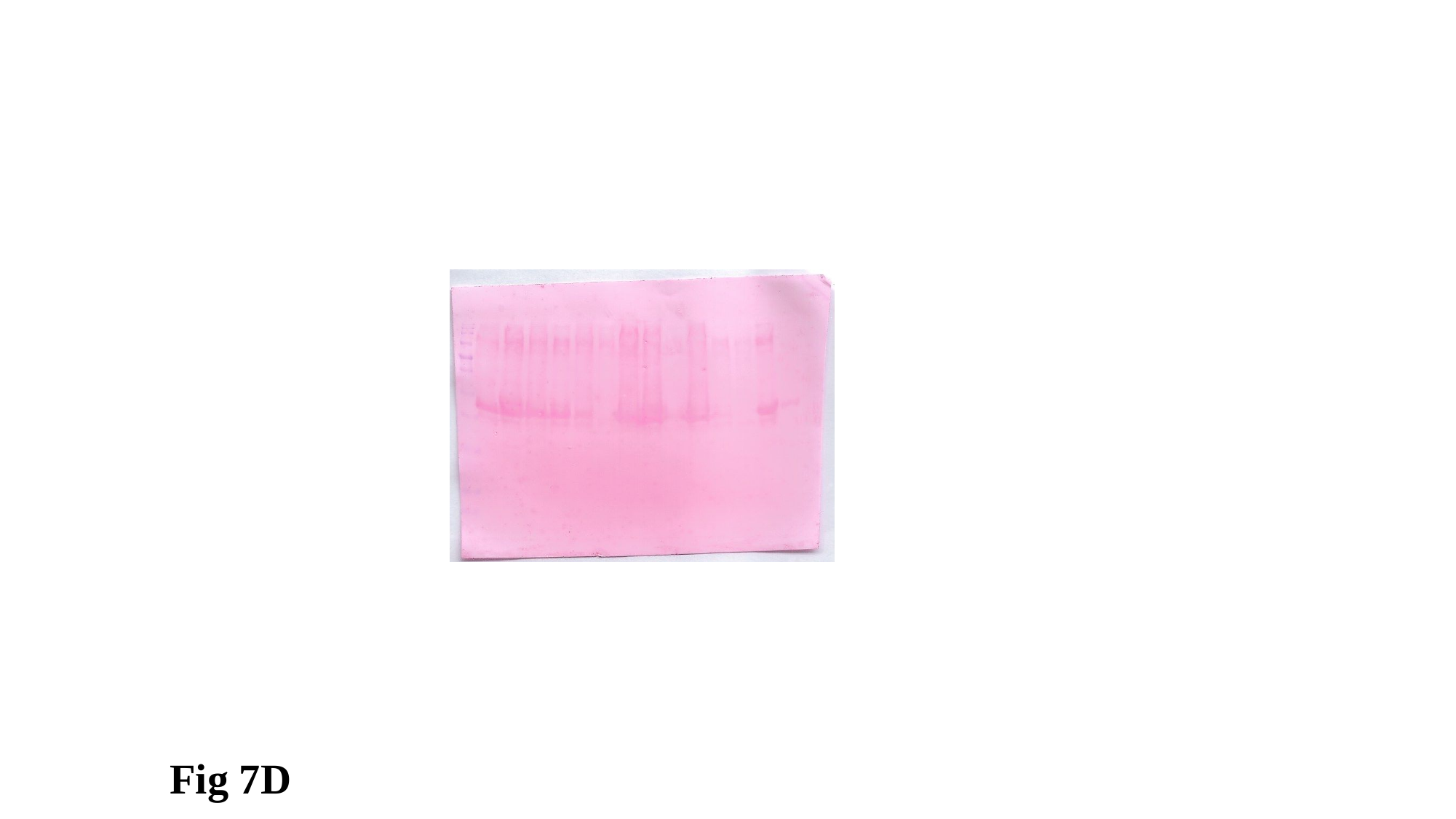

Fig 7D

## Slide 15
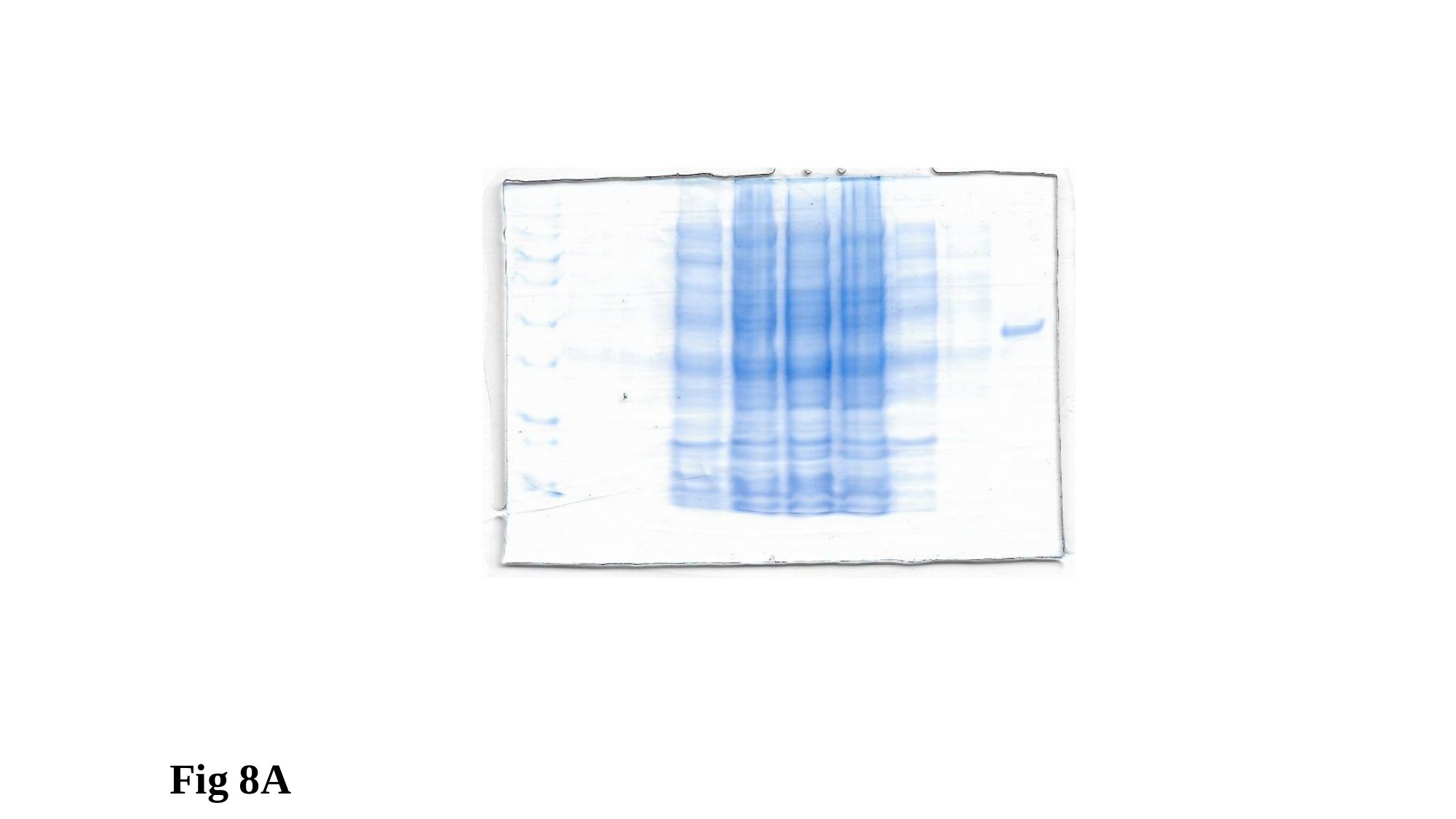

Fig 8A

## Slide 16
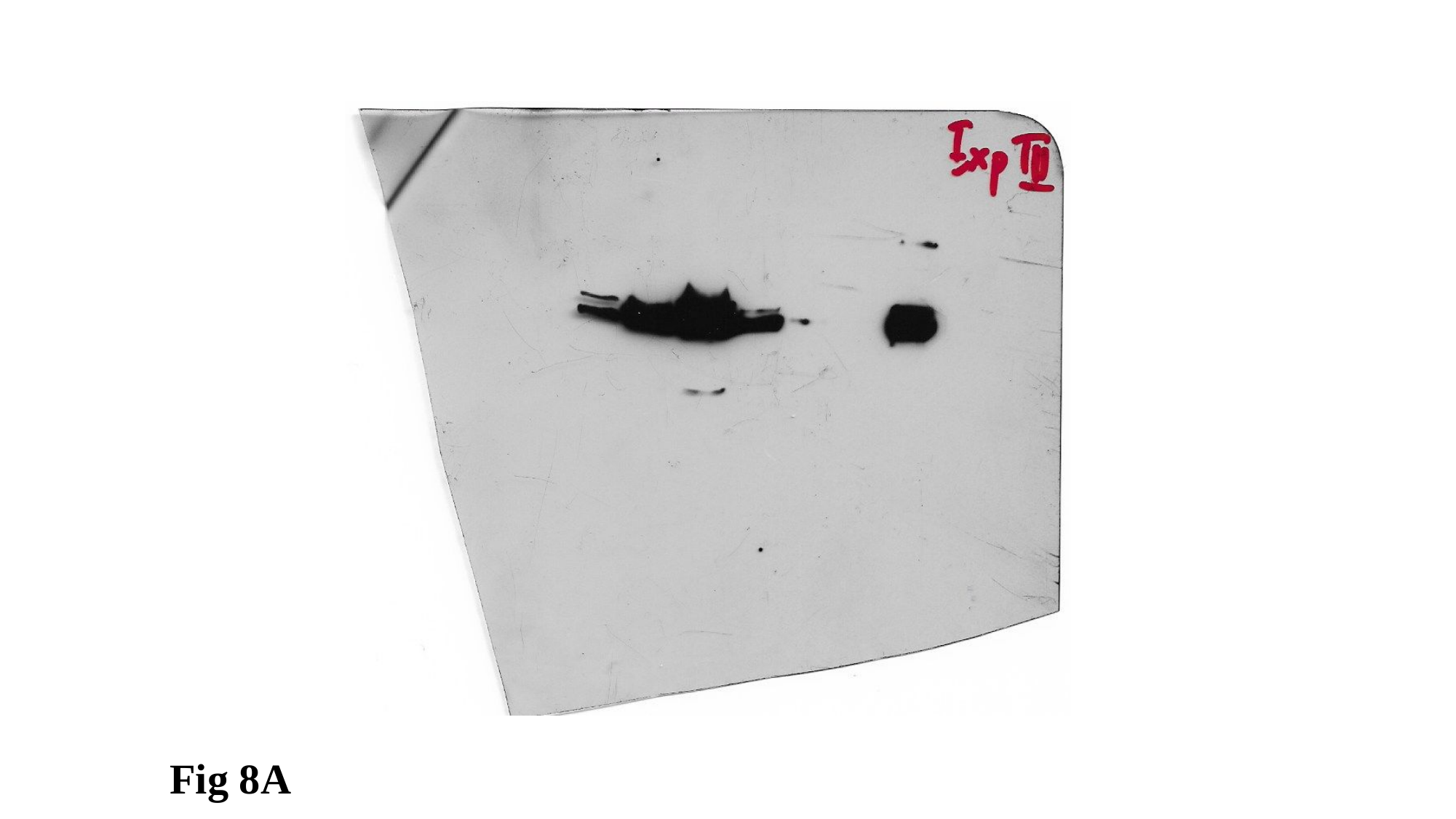

Fig 8A

## Slide 17
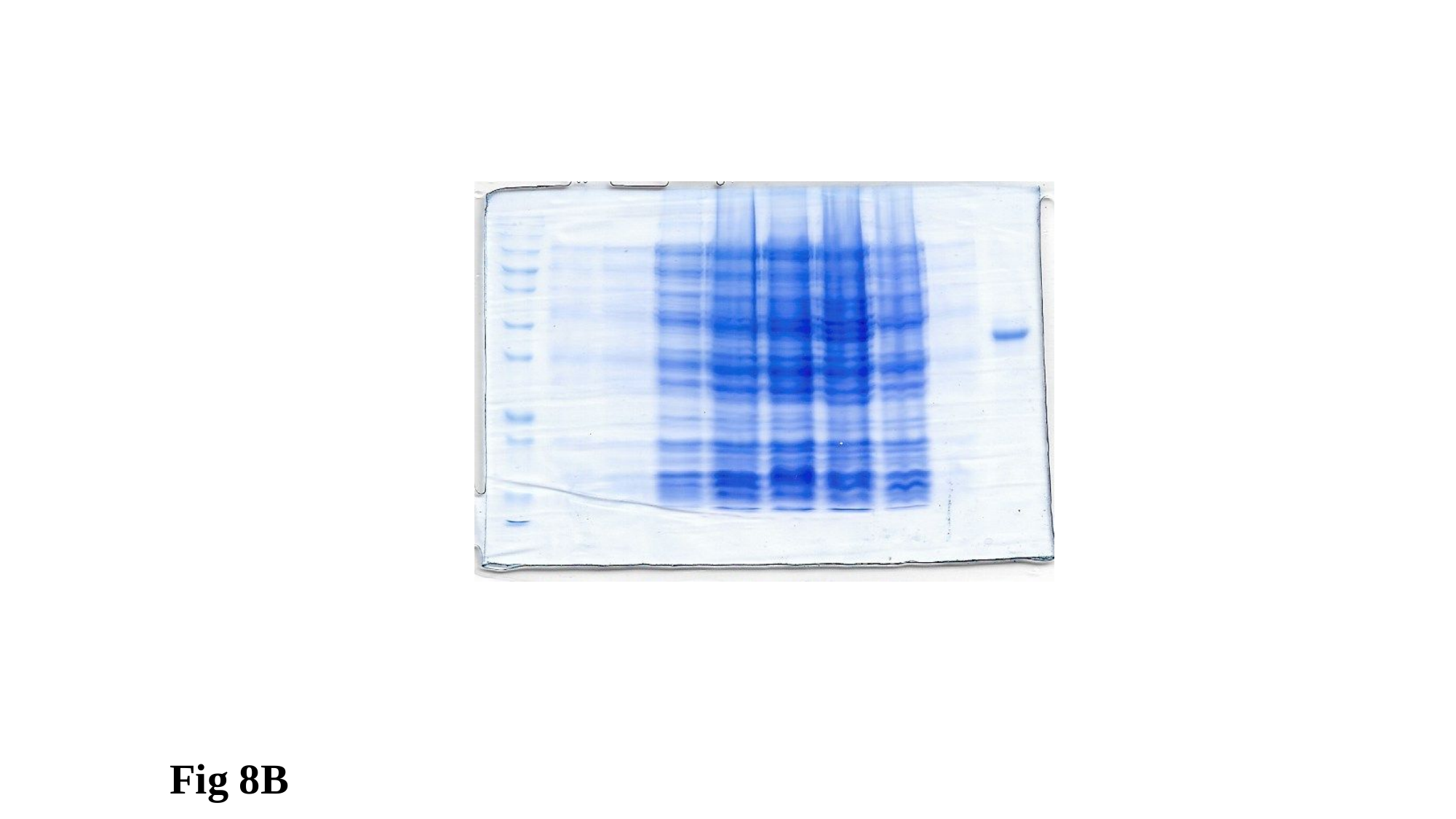

Fig 8B

## Slide 18
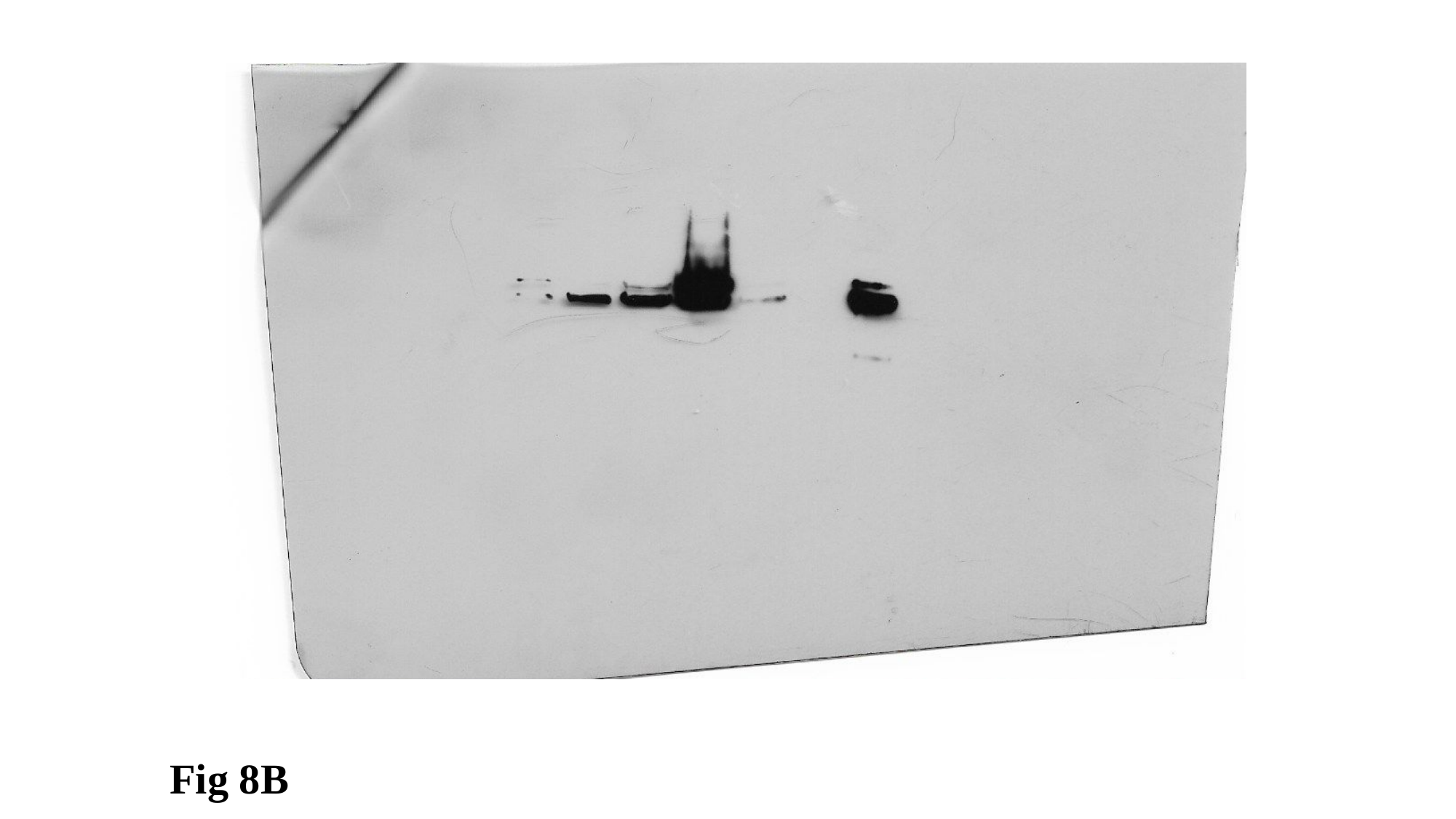

Fig 8B
