# Supplementary material for: Outer membrane protein N expressed in Gram-negative bacterial strain of Escherichia coli BL21 (DE3) Omp8 Rosetta strains under osmoregulation by salts, sugars, and pHs
Source: PLoS One. 2023 Aug 3;18(8):e0288096. doi: 10.1371/journal.pone.0288096 (PMC10399875; doi:10.1371/journal.pone.0288096)
Supplement: S1 Table — (DOC) [file pone.0288096.s003.doc]

**S1 Table. The pH values of LB broth medium containing monovalent and divalent cations was present.**

| **Salt concentrations**  **(Molar, M)** | **The pH values (±SD)** | | | | | | | |
| --- | --- | --- | --- | --- | --- | --- | --- | --- |
|  | **Monovalent cations** | | | | | **Divalent cations** | | |
|  | **LiCl** | **NaCl** | **KCl** | **RbCl** | **CsCl** | **MgCl_2_** | **CaCl_2_** | **BaCl_2_** |
| 0 | 6.65 ±0.07 | 6.64 ±0.03 | 6.30 ±0.03 | - | - | 6.62 ±0.07 | 6.52 ±0.03 | 6.66 ±0.03 |
| 0.025 | 6.48 ±0.02 | 6.51 ±0.02 | 6.31 ±0.01 | - | - | 6.32 ±0.02 | 5.98 ±0.02 | 6.28 ±0.01 |
| 0.05 | 6.40 ±0.06 | 6.47 ±0.02 | 6.29 ±0.02 | - | - | 6.17 ±0.06 | 5.97 ±0.02 | 6.13 ±0.02 |
| 0.075 | 6.32 | 6.43 ±0.03 | 6.30 ±0.02 | - | - | 6.07 | 5.88 ±0.03 | 6.06 ±0.02 |
| 0.1 | 6.31 ±0.01 | 6.38 ±0.04 | 6.29 ±0.02 | - | - | 6.03 ±0.01 | 5.81 ±0.04 | 5.97 ±0.02 |
| 0.25 | 6.14 ±0.03 | 6.26 | 6.25 ±0.02 | 6.45 ±0.20 | 6.52 ±0.09 | 6.77 ±0.03 | 5.61 | 5.80 ±0.02 |
| 0.5 | 5.99 ±0.02 | 6.21 ±0.02 | 6.21 ±0.01 | - | - | 5.50 ±0.02 | 5.35 ±0.02 | 5.54 ±0.01 |
| 0.75 | 5.84 ±0.04 | 6.15 ±0.02 | 6.20 ±0.02 | - | - | 5.39 ±0.04 | 5.21 ±0.02 | 5.38 ±0.02 |
| 1 | 5.71 ±0.02 | 6.10 ±0.03 | 6.20 ±0.01 | - | - | 5.22 ±0.02 | 5.07 ±0.03 | 5.28 ±0.01 |
| 1.5 | 5.71 ±0.02 | 6.01 ±0.01 | 6.18 | - | - | 4.99 ±0.02 | 4.77 ±0.01 | 5.01 |
| 2 | 5.58 ±0.02 | 5.94 | 6.18 | - | - | 4.77 ±0.02 | 4.48 | 4.86 |

The values shown are the results of tests that were carried out at least three times.

Standard deviation (±S.D.), Control is LB broth medium without any salts supplement (0 M), -; No experiment
