# Supplementary material for: Outer membrane protein N expressed in Gram-negative bacterial strain of Escherichia coli BL21 (DE3) Omp8 Rosetta strains under osmoregulation by salts, sugars, and pHs
Source: PLoS One. 2023 Aug 3;18(8):e0288096. doi: 10.1371/journal.pone.0288096 (PMC10399875; doi:10.1371/journal.pone.0288096)
Supplement: S2 Table — (DOCX) [file pone.0288096.s004.docx]

**S2 Table.** The NH_4_Cl, CH_3_COONa, CH_3_COONH_4_, Na_2_HPO_4_, NaH_2_PO_4_, and KH_2_PO_4_ components of LB broth medium were present, as well as the pH value.

| **Salt concentrations**  **(Molar, M)** | **The pH values (±SD)** | | | | | |
| --- | --- | --- | --- | --- | --- | --- |
|  | **NH_4_Cl** | **CH_3_COONa** | **CH_3_COONH_4_** | **Na_2_HPO_4_** | **NaH_2_PO_4_** | **KH_2_PO_4_** |
| 0 | 6.56 ±0.03 | 6.66 ±0.01 | 6.83 ±0.02 | 6.36 | 6.58 ±0.02 | 6.82 |
| 0.025 | 6.47 ±0.02 | 6.60 ±0.03 | 6.92 ±0.05 | 6.52 | 6.19 ±0.01 | 6.30 |
| 0.05 | 6.46 ±0.02 | 6.70 | 7.05 ±0.03 | 6.75 ±0.04 | 6.12 ±0.02 | 6.29 |
| 0.075 | 6.39 ±0.01 | 6.77 ±0.01 | 7.11 ±0.03 | 7.09 ±0.01 | 5.93 ±0.02 | 6.30 |
| 0.1 | 6.35 | 6.70 | 7.17 ±0.02 | 8.09 ±0.02 | 5.79 ±0.04 | 6.14 |
| 0.25 | 6.31 ±0.01 | 6.94 ±0.01 | 7.31 ±0.05 | 8.57 ±0.02 | 5.50 | 6.80 |
| 0.5 | 6.20 ±0.02 | 7.12 | 7.48 ±0.02 | 8.57 ±0.02 | 5.26 ±0.01 | 5.58 |
| 0.75 | 6.15 ±0.01 | 7.23 ±0.01 | 7.56 ±0.02 | 8.79 ±0.01 | 5.14 ±0.03 | 5.45 ±0.01 |
| 1 | 6.11 ±0.01 | 7.36 | 7.62 ±0.01 | 8.87 ±0.01 | 4.94 ±0.03 | 5.34 |
| 1.5 | 6.03 ±0.01 | 7.53 | 7.69 ±0.02 | 9.04 | 4.77 ±0.01 | 5.11 |
| 2 | 5.96 ±0.01 | 7.68 ±0.01 | 7.79 ±0.01 | 9.12 ±0.01 | 4.56 ±0.01 | 4.87 ±0.01 |

The values shown are the results of tests that were carried out at least three times.

Standard deviation (±S.D.), Control is LB broth medium without any salts supplement (0 M)
