# Supplementary material for: Outer membrane protein N expressed in Gram-negative bacterial strain of Escherichia coli BL21 (DE3) Omp8 Rosetta strains under osmoregulation by salts, sugars, and pHs
Source: PLoS One. 2023 Aug 3;18(8):e0288096. doi: 10.1371/journal.pone.0288096 (PMC10399875; doi:10.1371/journal.pone.0288096)
Supplement: Attachment — Submitted filename: Response to editor.doc [file pone.0288096.s005.doc]

Dear editor

Thank you for your comments,

Editor’s comment: Manuscripts are assessed for their suitability for consideration in PLOS ONE before entering the peer review process. Many more good articles are submitted to us for publication, and we give preference to those that present significant advances of broad scientific interest and significant impact in its field. Unfortunately, your manuscript has been declined at this stage, as it was not considered to meet these criteria. In addition, the abstract, introduction, and conclusions were poorly written and the entire manuscript run somewhat short of the standard threshold required for peer review consideration in its current form. However, the authors may kindly wish to improve the manuscript significantly and resubmit for consideration.

*Answer: The authors tried to improve the manuscript, following the abstract modification, introduction and also conclusion. Please see in the track changes version of the manuscript.*

Your sincerely,

Watcharin Chumjan
