# Supplementary material for: Outer membrane protein N expressed in Gram-negative bacterial strain of Escherichia coli BL21 (DE3) Omp8 Rosetta strains under osmoregulation by salts, sugars, and pHs
Source: PLoS One. 2023 Aug 3;18(8):e0288096. doi: 10.1371/journal.pone.0288096 (PMC10399875; doi:10.1371/journal.pone.0288096)
Supplement: Attachment — Submitted filename: PONE-D-23-08157.docx [file pone.0288096.s006.docx]

**Title:** EcOmpN expression in kanamycin antibiotic-resistant bacterial strains under osmoregulation by salts, sugars, and pHs

**Manuscript Number:** PONE-D-23-08157

**Short Title**: EcOmpN expression in kanamycin antibiotic-resistant bacterial strains

**Corresponding Author:** Watcharin - Chumjan, Ph.D. Rajamangala University of Technology Isan Khon kaen, Muang THAILAND

**OBSERVATIONS**

| **SN** | **TEXT LINE No.** | **COMMENTS** |
| --- | --- | --- |
| 1. | 90 | Correct the bacterial isolate specie name from “*Klebsiella pneumonia”* TO *Klebsiella* ***pneumoniae*** |
| 2. | 97 | The sentence “To create tryptone-medium preparation, LiCl, NaCl…….. was carried out.” **SHOULD** be moved to **MATERIALS and METHODS** instead of introduction |
| 3. | 128 | - “bacterial strains collected from Dr. Wipa Suginta…….” **SHOULD be used instead of “**bacterial strains were given by Dr Wipa Suginta,….” - There is a need for indicating how the bacterial strains were identified/confirmed microbiologically. If however the isolates were procured from culture collection center, then their identification number should be indicated. |
| 4. | 131 | The line “extract were used to make a BL-broth medium with a pH of 6.5.”  The media name should be **LB-broth medium** NOT BL-broth medium |
| 5. | 146-147 | The lines “….into a 50 ml Luria-Bertani medium (LB- broth) at 37^o^ C and then shaking for 18 hours at 200 rpm (overnight culture).” **SHOULD read**  “….into a 50 ml Luria-Bertani medium (LB- broth) **and incubated** at 37^o^ C **with regular** shaking for 18 hours at 200 rpm (overnight culture).” |
| 6. | 147-151 | This lines need to be reviewed to make the article technically sound as there is a gap in what the author is trying to show, the statement did not connect with one another.  “Various salts (XYZ) were designed and prepared at various concentrations (ABCD), called Luria Broth media and were also used to measure the pH values.” |
| 7. | 151 | The Line” The overnight culture incubation was then ………”  **SHOULD BE MODIFIED to**  The incubated overnight culture was then……… |
| 8. | 184 | After shaking all test tubes containing bacterial strains at 200 rpm (37^o^C) until the OD_600_ reached 0.6-0.7  **SHOULD BE MODIFIED to**  All test tubes containing bacterial strains were shaken at 200rpm and 37^o^C until the OD_600_ reached 0.6-0.7 |
| 9. | 226 | The heading should be modified e.g.:   - **Effect of salts and concentrations on the growth of gram-negative bacteria** |
| 10. | 449 | The conclusion need to be reviewed, What was written was more like the summary of what has been carried out rather than the conclusion |
| 11. | 458 | - The citation “Prilipov et al” **SHOULD** be made to conform to the journal standard (Numbered type). - There are two reference entries [26] and [27] all indicating Prilipov *et al*, WHICH among the two is the above citation (line 458) is referring to? |
| 12. | 743-756 | Tables 1 and 2 need to be make in a scientifically standard format, too much border lines between cells were used |
| 13. |  | Figure legends are too lengthy, too much information about method followed are included in the legend which are unnecessary there (e.g Fig legend 6, 7, 8 etc) |
| 14. |  | Tables and figures result need to show where they are statistically significant or otherwise |
| 15. |  | Tables need to have **Key** to indicate the meaning of symbols, acronyms and abbreviations. |
