# Supplementary material for: Outer membrane protein N expressed in Gram-negative bacterial strain of Escherichia coli BL21 (DE3) Omp8 Rosetta strains under osmoregulation by salts, sugars, and pHs
Source: PLoS One. 2023 Aug 3;18(8):e0288096. doi: 10.1371/journal.pone.0288096 (PMC10399875; doi:10.1371/journal.pone.0288096)
Supplement: Attachment — Submitted filename: EcOmpN expression in kanamycin antibiotic-resistant bacterial strains under osmoregulation by salts, sugars, and pHs.docx [file pone.0288096.s007.docx]

This manuscript reports the results of a laboratory research investigating the growth and expression of the outer membrane protein N (OmpN) in *E. coli* BL21 (DE3) Omp8 Rosetta under osmotic stress conditions caused by different salts, sugars, and pHs. I recommend the issues below to be tackled.

1) The title uses ‘kanamycin antibiotic-resistant bacterial strains’ while in the manuscript only *E. coli* BL21 (DE3) Omp8 Rosetta is mentioned. This may cause confusion for readers expecting information about several strains. Therefore, for coherence, the authors should either provide information about other strains or revise the title to reflect the focus on only one strain.

2) ‘EcOmpN’ is not a standard protein name and should be changed to ‘OmpN’ throughout the text*,* as it is the correct short form for the outer membrane protein N.

3) In line 31, it should be ‘tryptone yeast extract’ instead of ‘tryptone yeast’.

4) In lines 32-33, ‘the optical density at 600 nm was evaluated’ should be changed to ‘the optical density at 600 nm was measured’.

5) In line 34, ‘all salts’ is ambiguous.

6) In line 37-38, ‘Following the presence of the plasmid pEt21b(+)/ompN-BOR in these bacterial strains, which was expressed in the recombinant BOR protein’ it's not clear what ‘these bacterial strains’ refers to. It should be clarified.

7) In line 38, ‘pEt21b(+)/ompN-BOR’ should be ‘pET21b(+)/ompN-BOR’.

8) In line 41, define the abbreviation of ‘IPTG’ when first appeared in the manuscript.

9) In line 44, ‘According to research, bacterial growth in addition to salts, sugars, and pHs influences how the EcOmpN protein is produced’ is confusing. It is unclear what research is being referred to.

10) In line 61, the authors should specify which type of bacteria was used.

11) In line 71 ‘There are variations in the genotypes’ is not necessary and can be deleted.

12) In line 90,’ *Klebsiella pneumonia*’ should be ‘*Klebsiella pneumoniae*.’

13) In line 91, ‘which increases the OmpK36’ should be ‘which increases the expression of OmpK36.’

14) In line 97, ‘To create the tryptone-medium preparation, LiCl, NaCl, KCl, RbCl, CsCl, MgCl2, CaCl2, BaCl2, Na2HPO4, NaH2PO4, and KH2PO4 were used’ is unnecessarily long and redundant.

15) In line 131, ‘BL-broth’ should be written as ‘LB broth’ as it stands for Luria Bertani broth.

16) In line 133, the chemical ‘yeast’ should be ‘yeast extract.’

17) In line 155, it is not clear what ‘left by assessment’ means.

18) In line 158, the volume of glucose and fructose added to each tube should be mentioned.

19) In line 164, instead of ‘including pH 4, 5, 6, 7, 8, 9, and 10,’ it should be changed to ‘ranging from pH 4 to 10.’

20) In line 180, the use of ‘carried’ after ‘Rosetta’ is redundant.

21) In line 193, the type of bacterial cells used should be mentioned.

22) In line 211, it is not clear which groups were compared using the t-test. The names of the groups being compared should be spell out clearly.

23) In line 214, the use of ***P <0.0001 to evaluate statistical significance is incorrect, should be ***P <0.001.

24) In lines 232-247 and figures 1A, 1B, 2A and 2B, the text and figures described the growth of bacterial strains at varying salt concentrations, however no statistical analysis (P value) is mentioned to support the conclusions.

25) Line 257, seems like other strains apart from *E. coli* BL21 (DE3) Omp8 Rosetta were also used. Need to be clarified to avoid confusion for the readers.

26) In lines 266-267, should specify what concentration and type of salts were used.

27) In line 267, ‘With CH3COONa, a decrease in bacterial growth was noted’ is confusing because it does not specify what was the initial bacterial growth rate, or what was the control used for comparison.

28) In line 368, ‘Additionally, we noticed that the same bacteria strains, with the exception of CH3COONa and NH4Cl, could not grow at all on LB-medium containing CH3COONH4.’ This statement is vague, in addition the correct term is ‘bacterial strains,’ not ‘bacteria strains.’

29) In line 373, ‘Like the chlorides of MgCl2, CaCl2, and BaCl2 in group 2A, high bacterial growth was noted at 0.1 M, but not at 0.25 M, as was the case for the chlorides of other salts from other metals in group 1A.’ This statement needs to be clarified; the introduction of "group 2A" and "group 1A" here is very confusing.

30) In line 380, the use of ‘other’ is redundant and should be removed.

31) In line 386, ‘which was also seen’ is redundant and can be deleted.

32) In 402, ‘Reduced bacterial growth as a result of the reduced pH led to little or no EcOmpN expression’ is unclear and should be improved.

33) In lines 406-407, ‘The two of them, together with PhoE, were found in *E. coli* K-12’, it is not clear whether ‘them’ refers to the OmpF and OmpC genes or the OmpF and OmpC proteins.

34) In line 417, it is not clear whose findings the authors being referred to because the statement is not properly supported by the cited references. The authors should specify whose findings they are comparing their results to.

35) In line 456, the word ‘and’ seems to be incomplete.

36) In line 464, ‘The authors finding indicated’ should be changed to ‘The authors' findings indicated’
