# Supplementary material for: Outer membrane protein N expressed in Gram-negative bacterial strain of Escherichia coli BL21 (DE3) Omp8 Rosetta strains under osmoregulation by salts, sugars, and pHs
Source: PLoS One. 2023 Aug 3;18(8):e0288096. doi: 10.1371/journal.pone.0288096 (PMC10399875; doi:10.1371/journal.pone.0288096)
Supplement: Attachment — Submitted filename: Respond to reviewers.doc [file pone.0288096.s008.doc]

**Title:** EcOmpN expression in kanamycin antibiotic-resistant bacterial strains under osmoregulation by salts, sugars, and pHs

*Outer membrane protein N expressed in Gram-negative bacterial strain of Escherichia coli BL21 (DE3) Omp8 Rosetta strains under osmoregulation by salts, sugars, and pHs*

**Manuscript Number:** PONE-D-23-08157

**Short Title**: EcOmpN expression in kanamycin antibiotic-resistant bacterial strains

*Outer membrane protein N expressed in Gram-negative bacterial strain*

**Corresponding Author:** Watcharin - Chumjan, Ph.D. Rajamangala University of Technology Isan Khon kaen, Muang THAILAND

**OBSERVATIONS**

*Answers to observations in the below.*

| **SN** | **TEXT LINE No.** | **COMMENTS** |
| --- | --- | --- |
| 1. | 90 | Correct the bacterial isolate specie name from “*Klebsiella pneumonia”* TO *Klebsiella* ***pneumoniae***  *Answer: Yes, we changed.* |
| 2. | 97 | The sentence “To create tryptone-medium preparation, LiCl, NaCl…….. was carried out.” **SHOULD** be moved to **MATERIALS and METHODS** instead of introduction  *Answer: Yes, we moved it.* |
| 3. | 128 | - “bacterial strains collected from Dr. Wipa Suginta…….” **SHOULD be used instead of “**bacterial strains were given by Dr Wipa Suginta,….” - There is a need for indicating how the bacterial strains were identified/confirmed microbiologically. If however the isolates were procured from culture collection center, then their identification number should be indicated.   *Answer: Thank you for your comment here on the bacterial strains. But we would like to use this sentence*  *“The E. coli BL21 (DE3) Omp8 Rosetta bacterial strains were given by Dr Wipa Suginta, a professor at the Vidyasirimedhi Institute of Science and Technology (VISTEC) in Rayong, Thailand.*”  *Because this bacterial strain was normally used to produce the VhChiP as publication by Wipa, 2013 [24].* |
| 4. | 131 | The line “extract were used to make a BL-broth medium with a pH of 6.5.”  The media name should be **LB-broth medium** NOT BL-broth medium  *Answer: Yes, we corrected it and changed to LB broth medium as some to the comment of reviewer #2.* |
| 5. | 146-147 | The lines “….into a 50 ml Luria-Bertani medium (LB-broth) at 37^o^ C and then shaking for 18 hours at 200 rpm (overnight culture).” **SHOULD read**  “….into a 50 ml Luria-Bertani medium (LB- broth) **and incubated** at 37^o^ C **with regular** shaking for 18 hours at 200 rpm (overnight culture).”  *Answer: Yes, we modified it.*  *into a 50 ml LB broth medium and incubated at 37 ^o^C with regular shaking for 18 hours at 200 rpm (overnight culture).* |
| 6. | 147-151 | This lines need to be reviewed to make the article technically sound as there is a gap in what the author is trying to show, the statement did not connect with one another.  “Various salts (XYZ) were designed and prepared at various concentrations (ABCD), called Luria Broth media and were also used to measure the pH values.”  *Answer: Yes, we tried to add the sentence and modified it.*  *Numerous studies have demonstrated how the presence of NaCl in the medium affects bacterial growth [5]. Additionally, in vitro investigations utilizing black lipid membrane analyses have shown that various salts also impact the ion conductance of the outer membrane proteins [10, 14]. Thus, the author used various salts (such as LiCl, NaCl, KCl, RbCl, CsCl, MgCl_2_, BaCl_2_, CaCl_2_, NH_4_Cl, CH_3_COONa, Na_2_HPO_4_, NaH_2_PO_4_ and KH_2_PO_4_) to design and prepare the LB broth media at various concentrations (0, 0.025, 0.05, 0.075, 0.1, 0.25, 0.5, 0.75, 1, 1.5 and 2 M) and also measured the pH values of the medium.* |
| 7. | 151 | The Line” The overnight culture incubation was then ………”  **SHOULD BE MODIFIED to**  The incubated overnight culture was then………  *Answer: Yes, we modified it.*  *The incubated overnight culture was then pipetted and directly transferred to each of the test tubes containing the salts mentioned above, with a final optical density of 600 nm (OD_600nm_) equal to 0.1.* |
| 8. | 184 | After shaking all test tubes containing bacterial strains at 200 rpm (37^o^C) until the OD_600_ reached 0.6-0.7  **SHOULD BE MODIFIED to**  All test tubes containing bacterial strains were shaken at 200rpm and 37^o^C until the OD_600_ reached 0.6-0.7  *Answer: Yes, we modified it.*  *All test tubes containing bacterial strains were shaken at 200 rpm and 37 ^o^C until the OD600nm reached 0.6-0.7, 0.5 mM IPTG was added to each tube separately and then shaken continuously at 200 rpm for 3 hours at 20, 25, 30, 35, and 37 ^o^C.* |
| 9. | 226 | The heading should be modified e.g.:   - **Effect of salts and concentrations on the growth of gram-negative bacteria**   *Answer: Yes, we modified it.*  *The effect of salts and concentrations on the growth of Gram-negative bacterial growth* |
| 10. | 449 | The conclusion need to be reviewed, What was written was more like the summary of what has been carried out rather than the conclusion  *Answer: Yes, we did.* |
| 11. | 458 | - The citation “Prilipov et al” **SHOULD** be made to conform to the journal standard (Numbered type). - There are two reference entries [26] and [27] all indicating Prilipov *et al*, WHICH among the two is the above citation (line 458) is referring to?   *Answer: Yes, we referred to ref. 27 by Prilipov et al.* |
| 12. | 743-756 | Tables 1 and 2 need to be make in a scientifically standard format, too much border lines between cells were used  *Answer: Yes, we did.* |
| 13. |  | Figure legends are too lengthy, too much information about method followed are included in the legend which are unnecessary there (e.g Fig legend 6, 7, 8 etc)  *Answer: Yes, we tried to digest it.* |
| 14. |  | Tables and figures result need to show where they are statistically significant or otherwise  *Answer: Yes, we did.* |
| 15. |  | Tables need to have **Key** to indicate the meaning of symbols, acronyms and abbreviations.  *Answer: Yes, we did.* |
| **Reviewer #1** | | |
| 16. |  | Q1. The data do not fully support the conclusion because the conclusion was poorly written which is more like summary of what has been done.  *Answer: Yes, we modified it.*  ***Conclusion***  *The E. coli BL21 (DE3) Omp8 Rosetta strain is a gram-negative bacterial strain resistant to the drug kanamycin. This strain is typically used for producing outer membrane proteins. It lacks OmpF, OmpC, OmpA, and LamB, four of the main porins.* *According to earlier studies by the author, this bacterial strain produces the outer membrane protein N and VhChiP from the chip gene in Vibrio harveyi [24].* *There is no evidence of the effect of osmotic stress on the other three factors such as salts, sugars, and pH. The isolation of OmpN from E. coli K-12 and functional characterisation by black lipid membranes were only shown in an in vitro investigation by Prilipov et al [27]. The outcomes demonstrated the cation selectivity of its channel conductance and its preference for cation over anion.* *Also, the liposome swelling assay shows that the channel prefers monosugars over disugars.* *An earlier study enabled the author to test the notion that osmotic stress from salts, sugars, and any pH affected OmpN expression in this bacterial strain.*  *The authors’ finding indicated that growth in varied salt supplements in an LB broth medium had an impact on the bacterial growth of the E. coli BL21 (DE3) Omp8 Rosetta strain. All salts tested, except for CaCl_2_, CH_3_COONH_4_, and NaH_2_PO_4_, had an impact on bacterial growth at 0.25 M, whereas CsCl inhibited bacterial growth at all doses. This demonstrated that bacterial growth is dependent on salt [5, 17], much as Bacillus cereus was dependent on NaCl [7]. To induce OmpN expression and identify it using anti-OmpN polyclonal antibodies under bacterial growth conditions, glucose and fructose were used. After the addition of either glucose or fructose for three hours, high OmpN expression was observed. Low OmpN expression was perceived in the absence of sugar stimulation. Additionally, OmpN expression on the outer membrane of the E. coli BL21 (DE3) Omp8 Rosetta strain was verified using recombinant BOR protein. All salts, with the exception of BaCl_2_, CH_3_COONa, and KH_2_PO_4_, showed high levels of OmpN expression after 0.5 mM IPTG induction for 3 hours. Another observation of recombinant BOR protein expression at different pHs was that it was not present at pHs 4 and 10, which included both NaCl and KCl in the LB broth medium. For NaCl, high OmpN expression levels were seen at pH 6 and 7, while KCl showed high OmpN expression levels at pH 8. All results lead to the conclusion that osmoregulation by the salts, sugars, and pHs studied influences bacterial growth as well as OmpN expression levels.* *Salt, sugar, and pH are necessary for OmpN and recombinant BOR protein expression. This information will be used by the author to do additional research on how ions affect the conductance of the OmpN channel [8-10, 14, 27, 46]. Additionally, the interaction of the recombinant OmpN protein with sugar or antibiotics using black lipid membranes will be investigated [24].* |
| 17. |  | Q2. Most of the result does not capture whether the analysis was significant or otherwise especially in tables and figures, indicating that can make the reader understand the validity of the data.  Answer: *Yes, we did it.* |
| 18. |  | Q3. The data points behind means were not provided, the attached files were just like what was presented in the tables i.e the Means and standard deviations  Answer: *Yes, we did it.* |
| 19. |  | Q4. Yes BUT there is a need for improvement in some certain sections (Indicated in the attached comment sheet) so that the manuscript can be more technically sound  Answer: *Yes, we modified it.* |
| **Reviewer #2**  This manuscript reports the results of a laboratory research investigating the growth and expression of the outer membrane protein N (OmpN) in *E. coli* BL21 (DE3) Omp8 Rosetta under osmotic stress conditions caused by different salts, sugars, and pHs. I recommend the issues below to be tackled. | | |
| 20. |  | The title uses ‘kanamycin antibiotic-resistant bacterial strains’ while in the manuscript only *E. coli* BL21 (DE3) Omp8 Rosetta is mentioned. This may cause confusion for readers expecting information about several strains. Therefore, for coherence, the authors should either provide information about other strains or revise the title to reflect the focus on only one strain.  *Answer: Yes, we modified it.*  *Outer membrane protein N expressed in Gram-negative bacterial strain of Escherichia coli BL21 (DE3) Omp8 Rosetta strains under osmoregulation by salts, sugars, and pHs* |
| 21. |  | 2) ‘*Ec*OmpN’ is not a standard protein name and should be changed to ‘OmpN’ throughout the text, as it is the correct short form for the outer membrane protein N.  *Answer: Yes, we changed it.* |
| 22. | 31 | 3) In line 31, it should be ‘tryptone yeast extract’ instead of ‘tryptone yeast’.  *Answer: Yes, we changed it.*  *Here, tryptone yeast extract with varied salts and concentrations was initially used to generate an LB broth medium.* |
| 23. | 32-33 | 4) In lines 32-33, ‘the optical density at 600 nm was evaluated’ should be changed to ‘the optical density at 600 nm was measured’.  *Answer: Yes, we changed it.*  *To show how salts and concentration affect bacterial growth, the optical density at 600 nm was measured.* |
| 24. | 34 | 5) In line 34, ‘all salts’ is ambiguous.  *Answer: Thank you, we tried to use this “all tested salts”.*  *Moreover, a Western blotting study revealed that OmpN overexpression was present in all tested salts after stimulation with both glucose and fructose after being treated individually with anti-OmpN and anti-histidine tag polyclonal antibodies on transferred nitrocellulose membrane containing crude OmpN.* |
| 25. | 37-38 | 6) In line 37-38, ‘Following the presence of the plasmid pEt21b(+)/*ompN*-BOR in these bacterial strains, which was expressed in the recombinant BOR protein’ it's not clear what ‘these bacterial strains’ refers to. It should be clarified.  *Answer: Yes, we clarified it.*  *Following the presence of the plasmid pET21b(+)/ompN-BOR into E. coli BL21 (DE3) Omp8 Rosetta strains, which was expressed in the recombinant OmpN protein (BOR), OmpN expression was demonstrated for all monovalent cations as well as MgCl_2_.* |
| 26. | 38 | 7) In line 38, ‘pEt21b(+)/*ompN*-BOR’ should be ‘pET21b(+)/*ompN*-BOR’.  *Answer: Yes, we changed it.*  pET21b(+)/*ompN*-BOR |
| 27. | 41 | 8) In line 41, define the abbreviation of ‘IPTG’ when first appeared in the manuscript.  *Answer: Yes, we added it.*  *All of the tested salts, except for BaCl_2_, NaH_2_PO_4_, and KH_2_PO_4_, showed overexpression of recombinant BOR after Isopropyl β-D-1-thiogalactopyranoside (IPTG) induction.* |
| 28. | 44 | 9) In line 44, ‘According to research, bacterial growth in addition to salts, sugars, and pHs influences how the *Ec*OmpN protein is produced’ is confusing. It is unclear what research is being referred to.  *Answer: Yes, we pointed out.*  *According to this research, bacterial growth in addition to salts, sugars, and pHs influences how the OmpN protein is produced.* |
| 29. | 61 | 10) In line 61, the authors should specify which type of bacteria was used.  *Answer: Thank you, we replaced the word “Bact. coli”.*  *The best concentration of bact. coli growth was found at 0.20 M, regardless of the NaCl content (0.05, 0.10, 0.20, 0.30, or 0.40 M) or different pH level [18].* |
| 30. | 71 | 11) In line 71 ‘There are variations in the genotypes’ is not necessary and can be deleted.  *Answer: Yes, we deleted it.*  *Each is a distinct bacterial strain. BL21 (DE3) Omp8 Rosetta is E. coli B^E^, whereas E. coli DH5α is E. coli K-12. In addition, all outer membrane proteins are expressed in E. coli BL21 (DE3) Omp8 Rosetta, with the exception of LamB, OmpF, OmpC, and OmpA because it lacks the genes required for producing those porins [26].* |
| 31. | 90 | 12) In line 90,’ Klebsiella pneumonia’ should be ‘Klebsiella pneumoniae.’  *Answer: Yes, we corrected it.*  *In Klebsiella pneumoniae, for example, high osmotic stress causes the KbvR to be upregulated, which increases the expression of OmpK36 [30].* |
| 32. | 91 | 13) In line 91, ‘which increases the OmpK36’ should be ‘which increases the expression of OmpK36.’  *Answer: Yes, we changed it.*  *In Klebsiella pneumoniae, for example, high osmotic stress causes the KbvR to be upregulated, which increases the expression of OmpK36 [30].* |
| 33. | 97 | 14) In line 97, ‘To create the tryptone-medium preparation, LiCl, NaCl, KCl, RbCl, CsCl, MgCl_2_, CaCl_2_, BaCl_2_, Na_2_HPO_4_, NaH_2_PO_4_, and KH_2_PO_4_ were used’ is unnecessarily long and redundant.  *Answer: Yes, we tried to delete the name of salts and replaced with various salts.*  *To create the tryptone-medium preparation, various salts were used.* |
| 34. | 131 | 15) In line 131, ‘BL-broth’ should be written as ‘LB broth’ as it stands for Luria Bertani broth.  *Answer: Yes, we changed it.* |
| 35. | 133 | 16) In line 133, the chemical ‘yeast’ should be ‘yeast extract.’  *Answer: Yes, we added it.*  *All chemicals including tryptone, yeast extract, peptone, glucose, fructose, lithium chloride (LiCl), potassium chloride (KCl), rubidium chloride (RbCl), caesium chloride (CsCl), magnesium chloride (MgCl_2_), barium chloride (BaCl_2_), calcium chloride (CaCl_2_), ammonium chloride (NH_4_Cl), sodium acetate (CH_3_COONa), disodium phosphate (Na_2_HPO_4_), sodium phosphate (NaH_2_PO_4_) and potassium phosphate (KH_2_PO_4_) were purchased from Titan Biotech Ltd., India, Vivantis, Malaysia and Sigma-Aldrich, Denmark.* |
| 36. | 155 | 17) In line 155, it is not clear what ‘left by assessment’ means.  *Answer: Yes, we meant that the starter culture lefts from measurement by spectrometer.* |
| 37. | 158 | 18) In line 158, the volume of glucose and fructose added to each tube should be mentioned.  *Answer: Yes, we added and modified the sentence.*  *Subsequently, each tube was filled with 125 µl of 1 M glucose and fructose to final concentration of 0.025 M and followed by shaking at 200 rpm for 3 hours.* |
| 38. | 164 | 19) In line 164, instead of ‘including pH 4, 5, 6, 7, 8, 9, and 10,’ it should be changed to ‘ranging from pH 4 to 10.’  *Answer: Yes, we changed it.*  *Briefly, LB broth media containing 0.25 M NaCl and KCl were made separately at different pH values, ranging from pH 4 to 10, with citric acid or Tris-base adjustments.* |
| 39. | 180 | 20) In line 180, the use of ‘carried’ after ‘Rosetta’ is redundant.  *Answer: Yes, we changed to harboring.*  *For temperature effect on OmpN expression, E. coli BL21 (DE3) Omp8 Rosetta harboring recombinant plasmid (pET21b(+)/ompN) was grown in a LB broth medium containing ampicillin (100 µg/µl), kanamycin (25 µg/µl) and 1% glucose and shaken at 200 rpm for 18 hours at 37 ^o^C.* |
| 40. | 193 | 21) In line 193, the type of bacterial cells used should be mentioned.  *Answer: Yes, we added the name of bacterial strain.*  *The E. coli BL21 (DE3) Omp8 Rosetta strains containing the OmpN and recombinant OmpN protein were separated on a 12 per cent SDS-PAGE gel.* |
| 41. | 211 | 22) In line 211, it is not clear which groups were compared using the t-test. The names of the groups being compared should be spell out clearly.  *Answer: Yes, we tried to explain and re-wrote.*  *A statistical analysis that compared groups of tests for bacterial growth, such as E. coli BL21 (DE3) Omp8 Rosetta strains grown in tryptone-yeast medium prepared with various salts supplement versus no salts addition in tryptone yeast medium as a control, was done using the unpaired t-test.* |
| 42. | 214 | 23) In line 214, the use of ***P <0.0001 to evaluate statistical significance is incorrect, should be ***P <0.001.  *Answer: Yes, we corrected it.* |
| 43. | 232-247 | 24) In lines 232-247 and figures 1A, 1B, 2A and 2B, the text and figures described the growth of bacterial strains at varying salt concentrations, however no statistical analysis (P value) is mentioned to support the conclusions.  *Answer: Yes, we did it.* |
| 44. | 257 | 25) Line 257, seems like other strains apart from E. coli BL21 (DE3) Omp8 Rosetta were also used. Need to be clarified to avoid confusion for the readers.  *Answer: Thank you, we tried to indicate the bacterial strain used in this study.*  *The findings indicated that the E. coli BL21 (DE3) Omp8 Rosetta strains produced ammonia (NH_3_) as a by-product of the metabolism of carbon and nitrogen sources for the bacteria to live and raise the pH of the LB broth. High pH was observed for KCl during its optimal growth concentration range between 0.025 M and 0.75 M because it accelerated this bacterial growth (Table 2).* |
| 45. | 266-267 | 26) In lines 266-267, should specify what concentration and type of salts were used.  *Answer: Yes, we added it.*  *At four hours, the E. coli BL21 (DE3) Omp8 Rosetta starting culture was transferred into various salts with a growth concentration such as 0.25 M for LiCl, NaCl, KCl, RbCl, CH_3_COONa and NH_4_Cl; 0.1 M for MgCl_2_, BaCl_2_ and CaCl_2_; 0.075 M for Na_2_HPO_4_, NaH_2_PO_4_ and KH_2_PO_4_ (Fig. 4A).* |
| 46. | 267 | 27) In line 267, ‘With CH_3_COONa, a decrease in bacterial growth was noted’ is confusing because it does not specify what was the initial bacterial growth rate, or what was the control used for comparison.  *Answer: Yes, we added a control and re-wrote a new sentence.*  *In comparison to the positive control (LB broth medium containing 0.17 M NaCl), CH_3_COONa caused a reduction in bacterial growth.* |
| 47. | 368 | 28) In line 368, ‘Additionally, we noticed that the same bacteria strains, with the exception of CH_3_COONa and NH_4_Cl, could not grow at all on LB-medium containing CH_3_COONH_4_.’ This statement is vague, in addition the correct term is ‘bacterial strains,’ not ‘bacteria strains.’  *Answer: Yes, we tried to replace with E. coli BL21 (DE3) Omp8 Rosetta strains.*  *Additionally, we noticed that the E. coli BL21 (DE3) Omp8 Rosetta strains, with the exception of CH_3_COONa and NH_4_Cl, could not grow at all on LB broth medium containing CH_3_COONH_4_.* |
| 48. | 373 | 29) In line 373, ‘Like the chlorides of MgCl_2_, CaCl_2_, and BaCl_2_ in group 2A, high bacterial growth was noted at 0.1 M, but not at 0.25 M, as was the case for the chlorides of other salts from other metals in group 1A.’ This statement needs to be clarified; the introduction of "group 2A" and "group 1A" here is very confusing.  *Answer: Yes, we deleted and used other wording to replace group 1A “monovalent metal ions”.*  *Like the chlorides of MgCl_2_, CaCl_2_, and BaCl_2_, high bacterial growth was noted at 0.1 M, but not at 0.25 M, as was the case for the chlorides of other salts from other monovalent metal ions.* |
| 49. | 380 | 30) In line 380, the use of ‘other’ is redundant and should be removed.  *Answer: Yes, we deleted it.*  *Other observations on the bioluminescence of the three luminous bacterial species under their growth conditions of two-dimensional diffusion gradients of NaCl and H^+^ concentration.* |
| 50. | 386 | 31) In line 386, ‘which was also seen’ is redundant and can be deleted.  *Answer: Yes, we deleted it.*  *Bacterial proliferation led to OmpN expression. OmpN expression was detected by Western blot analysis using an anti-OmpN polyclonal antibody.* |
| 51. | 402 | 32) In 402, ‘Reduced bacterial growth as a result of the reduced pH led to little or no *Ec*OmpN expression’ is unclear and should be improved.  *Answer: Yes, we tried to improve the sentence.*  *Low or absent OmpN expression was a result of decreased bacterial growth in response to lower and higher pH.* |
| 52. | 406-407 | 33) In lines 406-407, ‘The two of them, together with PhoE, were found in E. coli K-12’, it is not clear whether ‘them’ refers to the OmpF and OmpC genes or the OmpF and OmpC proteins.  *Answer: Yes, we referred to both OmpF and OmpC.*  *Both OmpF and OmpC, together with PhoE, were found in E. coli K-12 [20].* |
| 53. | 417 | 34) In line 417, it is not clear whose findings the authors being referred to because the statement is not properly supported by the cited references. The authors should specify whose findings they are comparing their results to.  *Answer: Yes, we compared between in vitro and in vivo studies.*  *In in vitro investigations, CsCl was found to have a substantial ion conductance of OmpF in a series of LiCl, NaCl, and CsCl that was contained in a buffer at 0.1 M, but LiCl had a very low ion conductance [8]. Similar to our findings in vivo investigations, monovalent cations showed a significant contribution to bacterial growth while divalent cations worked as a restriction to inhibit bacterial growth.* |
| 54. | 456 | 35) In line 456, the word ‘and’ seems to be incomplete.  *Answer: Thank you, we tried to complete the sentence.*  *There is no evidence of the effect of osmotic stress on the other three factors such as salts, sugars, and pH.* |
| 55. | 464 | 36) In line 464, ‘The authors finding indicated’ should be changed to ‘The authors' findings indicated’  *Answer: Yes, we added it.*  *The authors’ finding indicated that growth in varied salt supplements in an LB broth medium had an impact on the bacterial growth of the E. coli BL21 (DE3) Omp8 Rosetta strain.* |
| **Reviewer #3**  This manuscript requires more extensive revisions because it is very limited in its scope and presentation of its results. This already starts on the title page, where the reader does not see that the authors’ university is located in Thailand. While this is merely a minor carelessness error, this style of negligence continues in the manuscript. For example, the abstract and the introduction promptly begin with a highly complex topic that is not introduced in detail. Neither the work’s relevance nor motivation becomes evident as a result. | | |
| 56. |  | Similarly, the abstract ends with a result without further statements about what conclusions follow from it. How this work, in turn, can be embedded in the state of research remains to be mentioned. In addition, there are, again, minor typographical errors, of which only a few can be listed. For example, Gram should be capitalized because this goes back to the name of a scientist. In the methods section, Rayong is mentioned without naming the country in which it is located. Also, typographically, there would be some things to improve. For example, the letter “x” should not be used as a substitute for the character identifying the cross product. In addition, variables should be italicized, as in the t-test, and numbers up to 12 should be written out as words. In the list of references, some terms in continuous capital letters crept in by mistake. In summary, this reviewer suggests a vast improvement of the manuscript to interest a readership beyond the very specialized studies.  *Answer: Thank you for your comments, we tried to improve the manuscript according to the reviewer’s comments.* |
