# Supplementary material for: Outer membrane protein N expressed in Gram-negative bacterial strain of Escherichia coli BL21 (DE3) Omp8 Rosetta strains under osmoregulation by salts, sugars, and pHs
Source: PLoS One. 2023 Aug 3;18(8):e0288096. doi: 10.1371/journal.pone.0288096 (PMC10399875; doi:10.1371/journal.pone.0288096)
Supplement: Attachment — Submitted filename: PONE-D-23-08157_R1.docx [file pone.0288096.s009.docx]

**Title:** EcOmpN expression in kanamycin antibiotic-resistant bacterial strains under osmoregulation by salts, sugars, and pHs

**Manuscript Number:** PONE-D-23-08157_R1

**Short Title**: EcOmpN expression in kanamycin antibiotic-resistant bacterial strains

**Corresponding Author:** Watcharin - Chumjan, Ph.D. Rajamangala University of Technology Isan Khon kaen, Muang THAILAND

**OBSERVATIONS For The REVIEWED MANUSCRIPT**

| **SN** | **TEXT LINE No.** | **COMMENTS** |
| --- | --- | --- |
| 1. | 90 | Correction was **satisfactorily** addressed |
| 2. | 97-99 | **Acceptable** |
| 3. | (Previously 128)  Now Line 105 | Observation raised is YET to be addressed properly   - As per your response, you can retain the statement you indicated HOWEVER, **since the initial concern is about confirming the authenticity/identity of that bacterial strain** (*E. coli* BL21 (DE3) Omp8 Rosetta) THEN you need to link your statement to that article e.g by adding “which was authenticated before Suginta *et. al* [24] (or any suitable reference number according to you write-up). - NOTE: in the same article you referred, Suginta et al 2013 **Referred** to the work of Prilipov et al 1998 [14] to verify the authenticity of the strain (**Which is more scientific in nature)** - Emulate the same way of confirming the authenticity as indicated above |
| 4. | (Previously 131)  (NOW line 108) | **Satisfactorily addressed** |
| 5. | (Previously 146-147)  Now 123-124) | **Satisfactorily addressed** |
| 6. | (Previously 147-151)  NOW 125-132 | Addressed **Satisfactorily** |
| 7. | (Previously 151)  NOW 132 | **Satisfactorily addressed** |
| 8. | (Previously 184)  NOW 166 | **Satisfactorily addressed** |
| 9. | Previously 226  NOW 225-226 | **Satisfactorily addressed** |
| 10. | (Previously 449)  NOW 525 | With the incorporated changes, **The conclusion is acceptable** |
| 11. | 458 | - **Satisfactorily addressed** |
| 12. | Previously 743-756.  NOW  297-327 | **Satisfactory** |
| 13. |  | **Concern was addressed in the figure shown** |
| 14. |  | - Acceptable |
| 15. |  | **Satisfactorily addressed** |
