# Supplementary material for: Outer membrane protein N expressed in Gram-negative bacterial strain of Escherichia coli BL21 (DE3) Omp8 Rosetta strains under osmoregulation by salts, sugars, and pHs
Source: PLoS One. 2023 Aug 3;18(8):e0288096. doi: 10.1371/journal.pone.0288096 (PMC10399875; doi:10.1371/journal.pone.0288096)
Supplement: Attachment — Submitted filename: 2nd Review.docx [file pone.0288096.s010.docx]

This manuscript reports the results of a laboratory research investigating the growth and expression of the outer membrane protein N (OmpN) in *E. coli* BL21 (DE3) Omp8 Rosetta under osmotic stress conditions caused by different salts, sugars, and pHs. I recommend that the article can be accepted if the following minor revisions are addressed.

In line 548, ‘’perceived’’ should be changed to ‘’observed’’

In line 552, ‘’different pHs’’ should be changed to ‘’different pH values.’’

In line 553, ‘’it was not present’’ should be changed to ‘’it was not detected.’’

In line 553, ‘’pHs 4 and 10’’ should be changed to ‘’pH values of 4 and 10’’, and the term ‘’pH’’ should be replaced with ‘’pH value’’ throughout the manuscript.

In line 553, ‘’included both NaCl and KCl in the LB broth medium’’ should be changed to ‘’when LB broth medium contained both NaCl and KCl.’’

In line 554, ‘’while KCl showed high OmpN expression levels’’ should be replaced with ‘’while high levels of OmpN expression were observed in the presence of KCl.’’
